# Chemical Profiling of Polyphenolic Fraction of *Cannabis sativa* L. vr. *Kompolti* Industrial Inflorescences: Insights into Cannabidiol Neuroprotective Effects in a Cellular Model of Parkinson’s Disease

**DOI:** 10.3390/plants14101473

**Published:** 2025-05-14

**Authors:** Francesca Fantasma, Gilda D’Urso, Noemi Martella, Alessandra Capuano, Eleonora Boccia, Vadym Samukha, Vincenzo De Felice, Gabriella Saviano, Federico Trombetta, Gianluigi Lauro, Marco Segatto, Maria Giovanna Chini, Giuseppe Bifulco, Agostino Casapullo, Maria Iorizzi

**Affiliations:** 1 Department of Biosciences and Territory, University of Molise, Contrada Fonte Lappone, 86090 Pesche, Italy; fantasma@unimol.it (F.F.); noemi.martella@unimol.it (N.M.); v.samukha@studenti.unimol.it (V.S.); defelice@unimol.it (V.D.F.); saviano@unimol.it (G.S.); marco.segatto@unimol.it (M.S.); iorizzi@unimol.it (M.I.); 2 Department of Pharmacy, University of Salerno, Via Giovanni Paolo II 132, 84084 Fisciano, Italy; gidurso@unisa.it (G.D.); acapuano@unisa.it (A.C.); eboccia@unisa.it (E.B.); glauro@unisa.it (G.L.); bifulco@unisa.it (G.B.); 3 Societa Cooperativa Agricola MarcheSana, Localita San Biagio 40, 61032 Fano, Italy; federico.trombetta@gmail.com

**Keywords:** *Cannabis sativa* L. vr. *Kompolti*, cannabinoids, phytochemicals, LC-MS/MS, antioxidant effect, Parkinson’s disease (PD), minerals, nutraceuticals, polyphenols, molecular docking

## Abstract

The ultra-high-performance liquid chromatography high-resolution mass spectrometry (LC-ESI-HR-MS/MS) technique was used to characterize the polyphenolic fraction of the hot water infusion (WI) of inflorescences of *Cannabis sativa* L. *Kompolti* variety, commercially used for food preparations or cosmetic purposes. On water infusion extract, we applied a multidisciplinary approach, where NMR, MS, in vitro cell-free and cell-based assays coupled with in silico studies, were used to rationalize at the molecular level the effects of the major component Cannabidiol (CBD), in a model of Parkinson’s disease (PD). The phytochemical analysis by LC-MS/MS led to the tentative identification of many components belonging to different classes of polyphenols, such as phenolic acids, flavonoids, and their glycosides. CBD and cannabidiolic acid (CBDA) were also detected in good amounts in the infusion, together with several minor cannabinoids. In addition, the water infusion WI was evaluated for mineral content, total phenolic content, flavonoid content, and antioxidant capacity by DPPH and FRAP methods. Notably, our results in a cellular model of PD highlight that CBD protects against rotenone-induced cell death without recovering neuronal morphology. These biological outcomes were rationalized by an in silico approach, where we hypothesize that CBD could influence the cellular response to oxidative stress via its interaction with the Keap1/Nrf2 pathway. In summary, these results enriched the nutraceutical profile of the water infusion of the inflorescences of the *Kompolti* cultivar, which demonstrated a high CBD content. This study could lead to the development of dietary supplements that could help in the management of clinical symptoms related to the antioxidant activity of CBD in the pathophysiology of PD, which remains poorly characterized.

## 1. Introduction

Neurodegenerative diseases such as Alzheimer’s disease (AD), Parkinson’s disease (PD), Huntington’s disease (HD), and multiple sclerosis (MS), as well as others, result in the deterioration of neurons in a progressive manner, leading to dementia, motor disorders, and further functional disabilities [1]. Neuronal degeneration is a global health challenge, increasing in developed societies and growing exponentially with age.

Increasing evidence sustains that oxidative stress is a crucial driver of cell death in several neurodegenerative conditions. Indeed, excessive accumulation of reactive oxygen species (ROS) causes damage to macromolecules and triggers detrimental cascades, leading to mitochondrial dysfunction and intracellular energy imbalance. Additionally, increased ROS contributes to the activation of signal transduction pathways involved in the production of pro-inflammatory mediators, promoting chronic neuroinflammation that exacerbates the disease phenotype [2]. Concurrently, pro-inflammatory cytokines enhance ROS production, creating a vicious cycle that contributes to the generation of oxidative stress in diseases such as AD, PD, HD, and MS [3,4,5].

Thus, new therapeutic approaches based on targeting oxidative stress and inflammatory components of neurological diseases need to be tested. In this context, several approaches have been explored, such as antioxidants, kinase inhibitors, immunotherapy, and repurposing existing medications [6,7]. Several phytocannabinoids isolated from *Cannabis sativa* have attracted attention as a potential therapeutic strategy in NDs. The Δ9-tetrahydrocannabinol (THC) is the main psychoactive component of *Cannabis*, while Cannabidiol (CBD), the main non-psychoactive component, has recently emerged as a potential prototype for drug development due to its antioxidant and anti-inflammatory properties and its well-tolerated pharmacological behaviour [8]. Cannabinoids are found in all aerial parts of the plant, accumulating mainly in glandular trichomes in leaves and bracts [9].

The study of THC led to the discovery of the endocannabinoid system (ECS), which regulates various physiological processes, such as cognition and analgesia, and acts as a molecular target in the central nervous system (CNS). ECS consists of (i) a collection of cannabinoid receptors recognized as CB1R and CB2R bodily expressed, (ii) two primary ECS ligands named anandamide (AEA) and 2-arachidonoylglycerol (2AG), and (iii) enzymes as fatty acid amide hydrolase (FAAH) and monoglyceride lipase (MAGL) that control the release and degradation of ECS [10].

Some cannabinoids can mimic the effect of endocannabinoids and can regulate neurotransmission in certain pathways [11]. CBD is not a primary ligand of CB1 or CB2, but it may influence their signalling by modifying endocannabinoid tone [12].

CBD has been found to have a variety of pharmacological effects that have applications in a range of conditions, including pain, cancer, inflammation, epilepsy, anxiety, and autism spectrum disorder. This suggests that CBD may interact with different molecular targets such as enzymes, nuclear receptors, ionotropic receptors, metabotropic receptors, etc. [13,14].

Most of the investigations have reported the anti-inflammatory effects of CBD through the reduction of pro-inflammatory cytokine synthesis [15,16]. In addition, it also shows antitumorigenic activity in both in vitro and in vivo models [17], improves motor activity, affects depression [18], reduces markers of inflammation in pancreas microcirculation in a Type 1 Diabetes mice model [19], and can reduce the anxiety and psychosis-like effects seen after THC administration [20].

CBD possesses intrinsic antioxidant effects [21,22], since it has the ability to donate electrons and inhibit lipid peroxidation, which inhibits caspase-mediated apoptosis of neurons [23].

Recently, CBD has attracted major attention due to its neuroprotective activity [24], its nonpsychoactive nature, and well-tolerated pharmacological behaviour [25,26,27].

The neuroprotective effect of CBD is stronger than conventional antioxidants such as α-tocopherol or ascorbate [28], as it prevents neuroinflammation by acting on multiple molecular targets [8]. Peroxisome proliferation-activated receptor gamma (PPARγ) [29,30] is an important target of CBD. Several studies have reported that PPAR-γ agonists exhibit neuroprotective effects via regulating gene transcription related to the pathogenesis of neurodegeneration [31]. Recently, the therapeutic potential of CBD has been linked to the nuclear factor erythroid 2-related factor 2 (Nrf2) signalling pathway. The relationship between Nrf2 and CBD is closely related to several proteins associated with cancer, neurodegenerative conditions, and cardiovascular dysfunction. In particular, Nrf2 is involved in the regulation of ROS metabolism in several physiopathological conditions, including neurodegeneration [32]. Because neurodegenerative diseases require long-term therapy, drugs must have a high safety profile and have access to specific target cells or pathways of the CSN to be clinically effective. CBD shows great promise due to its high safety profile and good tolerability. A breakthrough in *Cannabis* research came in 2005 with the approval of Sativex^®^, a combination of THC and CBD (1:1) for the treatment of spasticity and/or neuropathic pain in MS [33]. Clinical use of CBD alone began with the FDA approval of Epidiolex^®^ for the treatment of pediatric epilepsy in 2018 [34] and three synthetic cannabis-related drug products: Marinol^®^ (dronabinol), Syndros^®^ (dronabinol), and Cesamet^®^ (nabilone) [35]. Due to the impressive pharmacological profile of CBD and other non-psychoactive compounds, hemp extracts are often considered nutraceuticals for their health benefits. However, despite the wide range of biological effects attributed to CBD, the putative therapeutic role and antioxidant activity of CBD in the pathophysiology of PD remain poorly characterized.

Starting with these premises, the purpose of this study was multifaceted: (a) to determine the chemical composition of the polyphenolic fraction of WI extract of *C. sativa* L. *Kompolti* variety, (b) to define its in vitro antioxidant capacity using the DPPH and FRAP assays, (c) to determine its mineral composition, (d) and to isolate CBD as the major constituent of the WI extract for the next cell-based assays. Subsequently, (e) we investigated the effects induced by pure CBD in differentiated SH-SY5Y neuronal cells treated with rotenone toxin to induce the Parkinsonian phenotype. Finally, (f) after identifying Nrf2 as the most promising cellular partner for CBD, we used the in silico study to rationalize at the molecular level the observed biological activity.

## 2. Results and Discussion

### 2.1. Major Phytochemical Components in Kompolti Water Infusion

Medical *Cannabis* is legally available in several countries and has significant variations in phytocannabinoids content according to the cultivar and geographical area. Patients consume medical *Cannabis* in its dried form and in a variety of ways, including vaping, food preparation, or as infusions, herbal teas, decoctions, or infused or as edible oils [36]. The hot water extraction used in this study is a method to simulate the production process of *Cannabis* beverages, and the aqueous extract obtained is considered a beverage for human consumption. The first objective of the present study was to evaluate the chemical profile of the extract after infusion in hot water of *Kompolti* dried inflorescences. The hot WI was submitted to the modified Kupchan’s partitioning procedure [37], and four extracts were obtained (*n*-hexane, CHCl_3_, *n*-BuOH, and water residual extract), allowing better identification of components according to its polarity. Each extract was submitted to ^1^H-NMR (see Material and Methods) and LC-ESI-HR-MS/MS analysis.

#### Identification of Major Components in *C. sativa* by LC-ESI-HR-MS/MS Analysis

**Figure 1:**
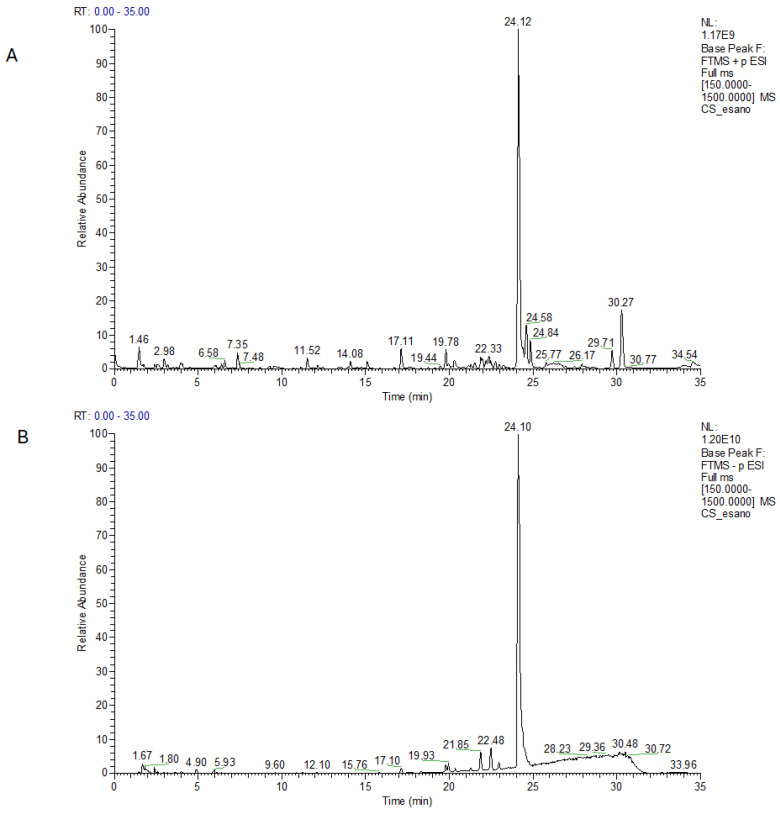
LC-MS profile of *n*-hexane extract in positive (**A**) and in negative ion mode (**B**).

Polyphenols are abundant in *C. sativa*, along with prenylated flavonoids, phenolic amides, and lignan amides. These metabolites are typical of *Cannabis* and are involved in the plant defense mechanisms. In particular, they act as antioxidants, preventing the formation of reactive oxygen species (ROS) [38,39]. In the *n*-hexane extract of WI, the ^1^H NMR experiment revealed a mixture of fatty acids and good amounts of cannabinoid derivatives (Supplementary Materials Figure S1). This was also supported by the LC-MS profiles in negative and positive ion modes (Figure 1A,B), which showed one main peak at a retention time 24.1 min and a few smaller peaks. Thanks to high-resolution mass spectrometry and MS/MS analysis combined with research in databases such as Compound Discoverer, Knapsack database (https://www.knapsackfamily.com/knapsack_core/top.php) (accessed on 1 February 2025) [40], and literature data refined on *C. sativa* species, peaks were putatively characterized as CBDA, CBD and lipids all listed in Table 1 according to their elution time.

Phytocannabinoids, which are the major constituents of *C*. *sativa*, are well-known terpenophenolic compounds, conventionally classified into different subclasses based on their chemical structures [41]. While these compounds share similar chemical formulas and exact masses, their structures and fragmentation patterns differ. Therefore, LC-MS/MS analysis is a valuable analytical technique widely used for detecting a broad range of these metabolites [41].

A predominance of the cannabinoids CBDA and CBD was observed in the ^1^H NMR spectrum of the *n*-hexane extract (Supplementary Materials, Figure S1), as evidenced by LC-MS analysis (Table 1). The assignments of selected CBD and CBDA signals in the ^1^H NMR spectrum are given in the Supplementary Materials (Figure S1) and are based on the current literature [42]. Much more interesting were the ^1^H NMR spectra of chloroform extract (Supplementary Materials, Figure S2), which showed a good content of cannabinoid derivatives, also confirmed by LC-MS.

**Figure 2:**
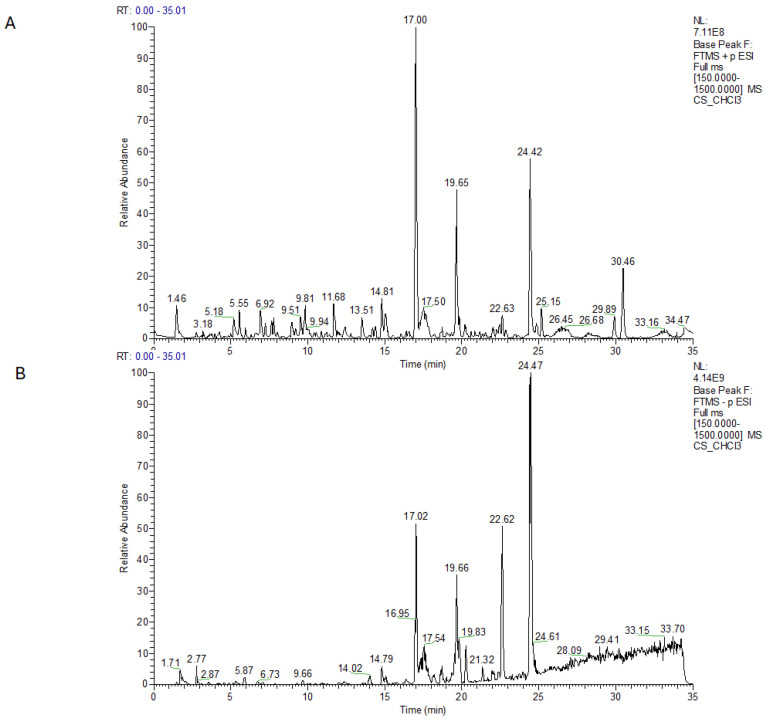
LC-MS profile of CHCl_3_ extract in positive (**A**) and in negative ion mode (**B**).

The LC-MS profile of the CHCl_3_ extract in both positive and negative ion modes (Figure 2A,B), exhibited the presence of four most intense peaks (Table 2). At 17 min retention times, two pseudomolecular ions were observed in the positive ion mode, one at *m/z* 375.2152 ([M + H]^+^) and at *m/z* 357.2048 ([M − H_2_O + H]^+^), while in the negative ion mode a pseudomolecular ion was detected at *m/z* 373.2013 ([M − H]^−^). These species converged to the molecular formula C_22_H_30_O_5_. A detailed analysis of the MS/MS spectra, in comparison with data from the literature [43], allowed the identification of this metabolite as cannabielsoic acid A (CBEA-A), belonging to the cannabielsoin subclass of phytocannabinoids. At retention times of 19.6 min and 24.4 min in positive ion mode, the same pseudomolecular ions at *m/z* 359.2204 were observed, suggesting a molecular formula of C_22_H_30_O_4_ for both. This formula could be attributed to cannabidiolic acid (CBDA), tetrahydrocannabinolic acid (THCA), or cannabichromenic acid (CBCA). Moreover, these ions showed the same MS/MS fragmentation pattern, with three major fragment ions at *m/z* 341.2105 corresponding to the loss of H_2_O, 261.1480, and 219.1014, which result from the breakage of the terpene moiety at the C1–C6 bond and from the loss of the terpene, respectively [43]. They were identified as CBDA and CBCA, respectively, and the fragmentation spectra confirmed that they could not be THCA. In negative ion mode, a better-ionized pseudomolecular ion was observed at *m/z* 375.2172 ([M − H]^−^) under the peak with a retention time of 19.6 min, with a molecular formula of C_22_H_32_O_5_. From the MS/MS spectra, it was clear that this ion belonged to phytocannabinoids, and by comparing the data reported in the literature for *C. sativa*, it could be identified as 6,7-Epoxy-Cannabigerolic acid (6,7-Epoxy-CBGA) [43].

Additionally, the detailed analysis of LC-MS profiles of the CHCl_3_ extract highlighted the presence of other minor metabolites, all listed in Table 2, according to their elution time. The negative ion mode revealed the presence of two lipids such as 15,16-DiHODE, a hydroxylated fatty acid derived from linoleic acid, and 16-Hydroxyhexadecanoic acid, a hydroxylated fatty acid derived from palmitic acid.

**Figure 3:**
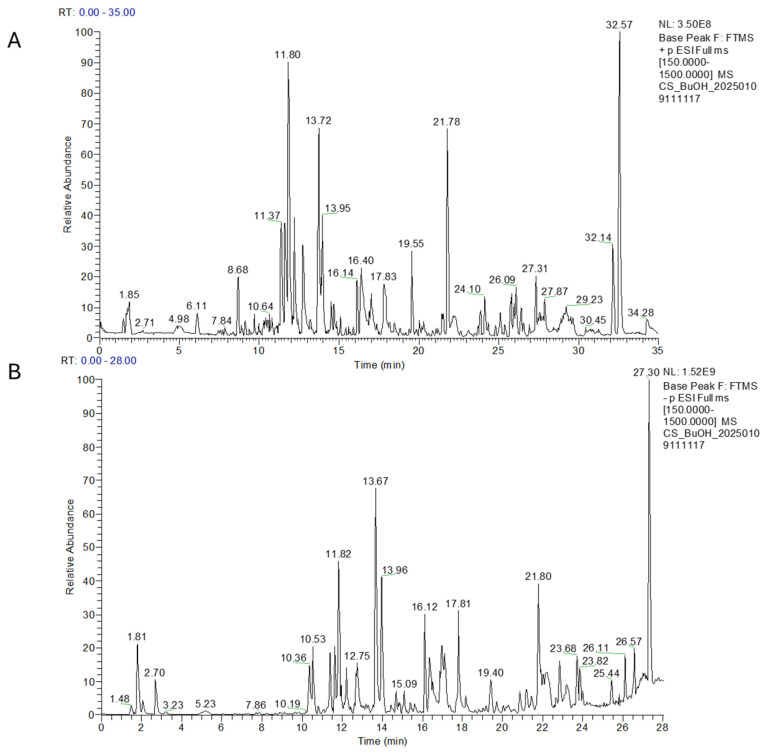
LC-MS profile of *n*-BuOH extract in positive (**A**) and in negative ion mode (**B**).

In the analysis of the ^1^H NMR spectrum (Supplementary Materials, Figure S3A,B), the *n*-BuOH extract was found to be very complex, rich in monosaccharide and polysaccharide components (3.0–5.0 ppm) and showed a mixture of polyphenols (6.0–8.5 ppm, Figure S3B). The LC-MS profiles (Figure 3A,B, and Table 3) were similarly highly complex, with several intense peaks, and the MS/MS analysis, combined with research in the literature, allowed the identification of various polyphenols along with different amino acids, both present in positive and negative ion mode. Apigenin, luteolin, and kaempferol derivatives were the most prevalent metabolites in this extract; traces of phytocannabinoids were also detected along with a diterpene-like andrographolide already reported in *C. sativa* [44,45].

**Figure 4:**
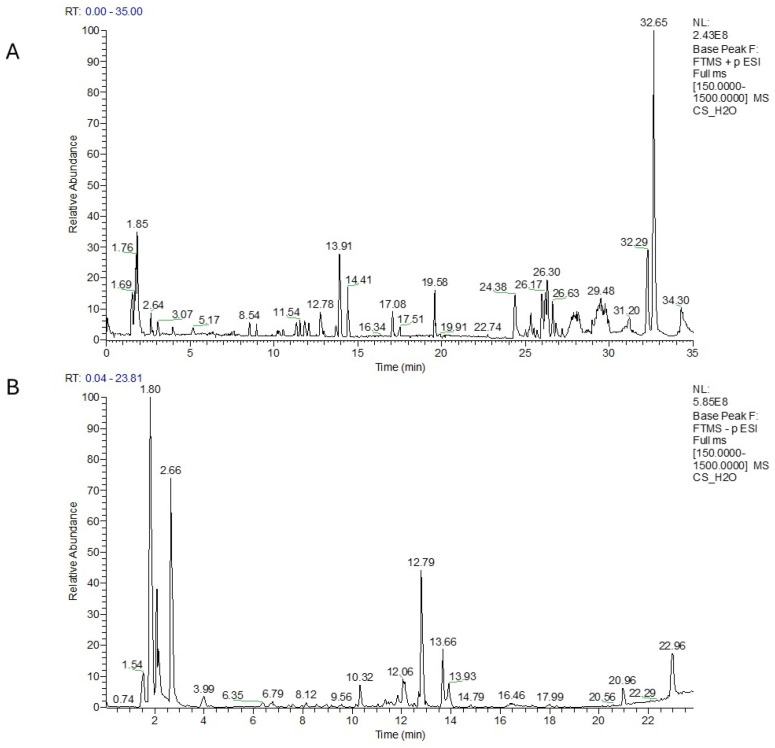
LC-MS profile of H_2_O extract in positive (**A**) and in negative ion mode (**B**).

Finally, in the ^1^H-NMR experiment, the main components detectable in the aqueous residue of WI (Supplementary Materials, Figure S4A–C), were mono- and polysaccharides (3.0–5.0 ppm), with a mixture consisting of small amounts of free amino acids (0.9–3.2 ppm) and organic acids (1.7–3.2 ppm). These data were confirmed by the LC-MS profile in positive and negative ion modes, which again revealed intense peaks at the beginning of the chromatography run. Thanks to the high resolution and the search in the database and the literature, these peaks were identified as amino acids, and sugar derivatives such as galactonic and galactaric acid (Figure 4A,B and Table 4). Phenolic acids such as protocatechuic acid hexoside and sinapoyl glucoside have also been identified in this extract, as well as some apigenin, kaempferol, and luteolin derivatives in glycosidic forms [46]. The major retention time in positive ion mode (Rt 32.57 min), was for octadeca-9,12-dienal, a metabolite previously reported in plant extracts such as ginger and olive oil [47,48].

Overall, a detailed analysis of the four extracts showed that the *n*-hexane and CHCl_3_ fractions exhibited higher phytocannabinoid content, with a predominance of CBDA and CBD (Supplementary Materials Figures S5–S7) and traces of polar lipids. The *n*-BuOH fraction was rich in phenolic acids and flavonoids, especially apigenin and luteolin in their glycosylated forms. Glycosylated flavonoids were also detected in traces in the aqueous residue, which mainly contained sugars and amino acids. Thus, we could confirm that the hot infusion of *Kompolti* inflorescences showed a complex metabolite composition, dominated primarily by primary plant metabolites such as carbohydrates and amino acids, but especially by phytocannabinoids and their derivatives, together with various phenolic compounds, which are secondary metabolites with significant nutraceutical and pharmaceutical value. Therefore, comprehensive chemical analyses such as those presented in the current study can help to facilitate the adoption by the medical community of products based on medical *Cannabis* in the form of water extracts.

### 2.2. Phenolic Components and In Vitro Antioxidant Activity

Polyphenols are a group of compounds with high diversity in means of functional groups such as phenolic terpenes, flavonoid aglycones, phenolic acids, and other highly methoxylated aglycone forms. They are usually soluble in polar organic solvents, and generally, but not always, their solubility in water increases with the number of hydroxyl groups or the presence of monosaccharide units. Structural differences between the various polyphenols affect their extractability and, thus, concentration and antioxidant activity in the extracts [49]. Cheng et al., 2023, showed that the concentration of antioxidants in beverages (water extracts) increases with elevation of the water temperature. At high temperatures, water polarity decreases, facilitating the solubilization of medium polarity compounds [50].

In our study, we also chose to prepare a hydroalcoholic extract of *C. sativa* (HEC) with MeOH/H_2_O (8:2) as solvent, a mixture commonly used to obtain cannabis extracts [51,52]. The aim was to compare the polyphenol content obtained by organic solvent extraction with the polyphenol level found in WI, which is the most environmentally friendly and traditionally used method of preparation.

The total phenolic content of both extracts, HEC and WI, was compared along with their antioxidant potential, and the results are summarized in Table 5.

The highest phenolic extraction yield was obtained with the 80% *v/v* methanol-water mixture, while the aqueous extract yielded a slightly lower concentration, with 3.29 mg GAE g^−1^ in HEC and 1.00 mg GAE g^−1^ in WI. This trend was also observed in the evaluation of flavonoid content in HEC (1.39 mg QUE g^−1^) and WI (0.48 mg QUE g^−1^). The evidence that the methanol-water solvent was more efficient is probably due to its intermediate polarity compared to water, which may promote the formation of more hydrogen bonds [53]. The results obtained are in line with those reported for aqueous extracts of other industrial hemp inflorescences by Ferrante et al. [54].

The antioxidant activity of both extracts was determined by in vitro DPPH and FRAP assays. They act by a different mechanism: DPPH is a synthetic radical used to measure the ability of ‘antioxidants’ to scavenge free radicals or donate electrons to quench the radical; in the FRAP assay, antioxidant capacity is determined by measuring the sample’s capacity to reduce the complex of ferric ions (Fe^3+^) to that of ferrous ions (Fe^2+^). In our samples, DPPH resulted in 58.87 (mg Kg^−1^ TE) in HEC, while 11.28 (mg Kg^−1^ TE d.w.) was observed in WI, the latter having a higher antiradical capacity (Table 5). In contrast, the HEC exhibited a significantly higher FRAP value (2.76 ± 0.03 mmol kg^−1^ TE d.w.) compared to the WI (1.49 ± 0.01 mmol kg^−1^ TE d.w.). These values denote a much higher antioxidant capacity of HEC in terms of reducing power.

### 2.3. Correlation Between Phenolic Compounds and Antioxidant Activity of Extracts

The relationship between total phenolics (TPC), total flavonoids (TFC), and antioxidant activity in extracts is not always direct. We found that higher concentrations of TPC and TFC in HEC corresponded to greater antioxidant activity only using the FRAP method. In contrast, WI showed greater effectiveness as an antioxidant by the DPPH method, despite the lower concentration of TPC and TFC. This apparent discrepancy could be explained by the difference in the composition of the HEC and WI extracts and by the different chemical mechanisms involved in the DPPH and the FRAP methods. As shown by HPLC-MS analysis, CBD, with its derivatives and various polyphenols (flavonoids, glycosylated flavonoids, phenolic acids, etc.), act as radical scavengers and were detected in higher concentrations in WI extract. In addition, CBD has also been reported to have antioxidant activity through hydroxyl groups of its phenolic ring [22]. CBD can influence the redox balance by altering the levels and activity of oxidants and antioxidants and disrupting free radical chain reactions by scavenging or converting them into less active forms [28].

In summary, the hydroalcoholic extract (HEC) showed a higher concentration of total polyphenols/flavonoids and superior antioxidant properties in FRAP tests, probably due to its higher concentration of low-polarity bioactive compounds. The *Cannabis* infusion (WI) showed better performance in the DPPH test, suggesting that it may contain compounds with high free radical scavenging capacity. However, the content of total polyphenols/flavonoids was lower.

These results highlight the complexity of antioxidant activity and the close relationship between the different mechanisms by which they may contribute to antioxidant effects.

### 2.4. Mineral Composition of the Extracts

The concentrations of macro- and microelements in *Cannabis* inflorescences and its infusions (WI) were determined by microwave-assisted digestion and using Inductively Coupled Plasma Optical Emission Spectroscopy (ICP-OES). The mineral content of the dried inflorescences (MCI) was also determined to compare the results that are summarized in Table 6. The evaluation of macroelements (i.e., P, K, Ca, Mg, S) and microelements revealed highly significant differences between mineralized *Cannabis* inflorescences (MCI) and WI, as each showed a unique mineral profile.

Among the macroelements, the MCI was found to possess a high calcium (Ca) content, with a concentration of 50.40 ± 0.24 g kg^−1^, in agreement with the results reported by Zafeiraki et al. [55], and at a higher concentration than WI (5.34 ± 0.09 g kg^−1^). This difference is probably due to the low solubility of this element in water, whereas other elements with higher solubility are extracted more effectively in aqueous media [56]. Potassium (K), magnesium (Mg), and phosphorus (P) were also detected in higher concentrations in MCI than in WI.

MCI and WI significantly differed in terms of the accumulation of microelements (i.e., Fe, Cu, Zn, B, Ba Co, Cr, Pb, V, and Ni). Iron (Fe), one of the most abundant trace elements in MCI, was quantified as 365.39 ± 37.03 mg kg^−1^, but only 2.86 ± 0.14 mg kg^−1^ was detected in WI. The significant decrease in Fe concentration in WI suggests that much of this metal is not readily transferred to the water-based infusion [57].

The same trend was observed for zinc (Zn) and boron (B), which showed a marked reduction from 58.41 ± 0.82 mg kg^−1^ (MCI) to 19.00 ± 0.11 mg kg^−1^ (WI) and from 66.91 ± 1.96 mg kg^−1^ (MCI) to 8.57 ± 0.08 mg kg^−1^ in WI. Other trace elements, including nickel (Ni), barium (Ba), cobalt (Co), chromium (Cr), lead (Pb), vanadium (V), zinc (Zn), and boron (B), showed low solubility in the aqueous infusion.

These differences further emphasize that while some macro and micro elements are effectively extracted into the hot infusion, others, particularly those bound to more complex cellular structures, are retained in the solid matrix due to the presence of proteins, fats, and fibers, which can reduce the solubility of metals. Differences in solubility and bioavailability of elements and extraction conditions such as temperature, pH, and solvent polarity may also affect mineral concentrations [58].

### 2.5. Effects of CBD from C. sativa L. in a Cellular Model of Parkinson’s Disease

**Figure 5:**
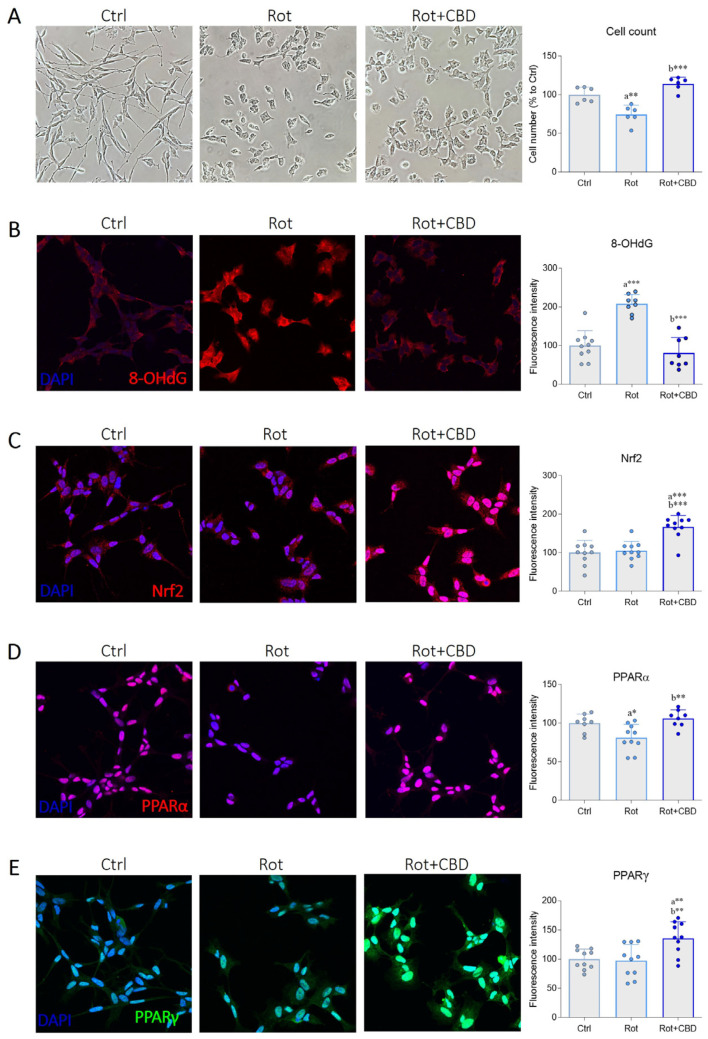
Effects of cannabidiol extracted from *Cannabis sativa* L. in a cellular model of Parkinson’s disease. (**A**) Bright field representative images and cell count of SH-SY5Y neuronal cell line. Differentiated cells were treated with vehicle (DMSO 0.001%, Ctrl) and rotenone (Rot, 0.1 μM) without or with cannabidiol (Rot + CBD, 1 μM). After 48 h, cells were fixed, and the images were captured. The graph represents the cell number for each experimental group (expressed as a percentage of the control) by analyzing six randomly selected fields. N = 6 biological replicates. (**B**–**E**) Immunofluorescence images and semi-quantitative analysis of (**B**) 8-OHdG (red), (**C**) Nrf2 (red), (**D**) PPARα (red), and (**E**) PPARγ (green) in SH-SY5Y cells treated as previously described. N = 8–10 independent experiments. DAPI was used to counterstain cell nuclei. Data are expressed as mean ± standard deviation (SD). The dots around the SD represent the different experimental values. Statistical analysis is carried out using one-way ANOVA tests and Tukey post hoc tests. Significance is indicated as follows: * *p* < 0.05; ** *p* < 0.01; *** *p* < 0.001. “a” indicates statistical significance vs. Ctrl group; “b” indicates statistical significance vs. Rot group.

The potential protective ability of CBD to counteract cytotoxic damage was investigated in the rotenone cell model of PD. As expected, bright-field images revealed a significant reduction in cell number after rotenone treatment compared to the control group (Figure 5A). Focusing on cell morphology, rotenone cells showed incontrovertible signs of toxicity, as reflected by altered cell body structure and a marked neurite retraction. Interestingly, co-treatment with CBD at a dose of 1 μM resulted in a significant reduction of cell death, although neurite extension was not prevented (Figure 5A). Since oxidative stress and redox imbalance play crucial roles in the uncontrolled progression of dopaminergic neurodegeneration of PD [59], the antioxidant ability of CBD was evaluated. Immunofluorescence and confocal analysis revealed that rotenone exposure led to a marked increase in immunoreactivity of 8-hydroxydeoxyguanosine (8-OHdG), an oxidized derivative of guanine commonly used as a marker of oxidative damage to nucleic acids. Notably, signal intensity was significantly reduced in the presence of CBD, indicating a protective effect of the cannabinoid against the oxidative stress induced by the environmental toxin (Figure 5B). Despite its intrinsic free radical scavenging ability, several experimental findings have highlighted that CBD efficiently influences redox balance by affecting the expression and activity of proteins belonging to the antioxidant machinery [60]. To explore the mechanisms mediated by CBD in reducing oxidative stress, we focused on the main cellular antioxidant pathways. Nrf2 is a key transcription factor that governs cellular defense against toxic and oxidative insults by promoting the expression of antioxidant genes [61]. While rotenone treatment did not affect Nrf2 expression, CBD increased the immunopositivity of the transcription factor in Parkinsonian cells, particularly at the nuclear level (Figure 5C). Besides Nrf2, the activity of peroxisome proliferator-activated receptors (PPARs) plays a crucial role in maintaining brain homeostasis. These nuclear receptors function as transcription factors that contribute to counteracting oxidative stress, enhancing mitochondrial function, and reducing neuroinflammation in dopaminergic neurons in the Substantia Nigra pars compacta (SNpc) and other brain regions [62]. Rotenone-induced ROS overproduction led to a reduction of PPARα expression in differentiated SH-SY5Y cells, which was effectively prevented by CBD treatment (Figure 5D). Differently, exposure to the neurotoxin did not affect PPARγ expression, while its immunoreactivity was significantly increased following CBD administration, resulting in a marked nuclear localization (Figure 5E). Our findings demonstrate that, although CBD does not prevent morphological changes of neurodegeneration, it exerts significant neuroprotective effects against rotenone-induced cytotoxicity by promoting cell viability. Overall, these results suggest that the antioxidant properties of CBD may be linked to its ability to enhance the relative abundance of key transcription factors involved in the antioxidant response, such as Nrf2, PPARα, and PPARγ. It is reasonable to hypothesize that the increased expression of transcription factors observed in this study could be attributed to the ability of CBD to promote their activation and/or expression [63]. Notably, several studies have identified CBD as a PPARγ agonist, and CBD-mediated PPARγ activation has been shown to elicit neuroprotective effects in various physiopathological contexts [63,64]. Additionally, recent molecular docking studies suggest a direct interaction between CBD and PPARα [65], although this possibility requires further investigation.

### 2.6. CBD/Nrf2 Complex: Molecular Docking Studies

CBD (Supplementary Materials Figure S5) is recognized to have antioxidant and anti-inflammatory properties [22], alongside the well-known interaction mechanisms between CBD and PPARα, and PPARγ (vide supra). To gain a clearer understanding of the potential interaction of CBD with Nrf2, we performed molecular docking calculations. Given the absence of full-length Nrf2 crystal structures (only small sequence fragments are available), we focused on assessing the potential interference of CBD with the Nrf2-Keap1 interaction. This choice was driven by the literature evidence demonstrating that Nrf2 binds to Keap1 [66] through two conserved motifs, the ETGE and DLG motifs [67], located within the Neh2 domain of Nrf2. These interactions are critical for Keap1-mediated ubiquitination and subsequent proteasomal degradation of Nrf2, thereby regulating its cellular levels. The disruption of the Keap1-Nrf2 interaction has been identified as a key mechanism for the activation of the Nrf2-dependent antioxidant response: some small molecules are able to bind the Kelch domain of Keap1, hindering its interaction with Nrf2 and, as a result, its degradation [68]. Based on this regulatory mechanism, we explored whether CBD could interact with the Keap1 binding pocket, potentially interfering with its ability to sequester and degrade Nrf2. Furthermore, studies on keratinocytes exposed to UV light have highlighted that the treatment with CBD can induce a reduction in Keap1 levels, suggesting an indirect modulation of Nrf2 activity [69]. This mechanism underlines the potential of CBD to modulate the cellular response to oxidative stress through the interaction with Keap1 and the activation of the Nrf2 pathway.

**Figure 6:**
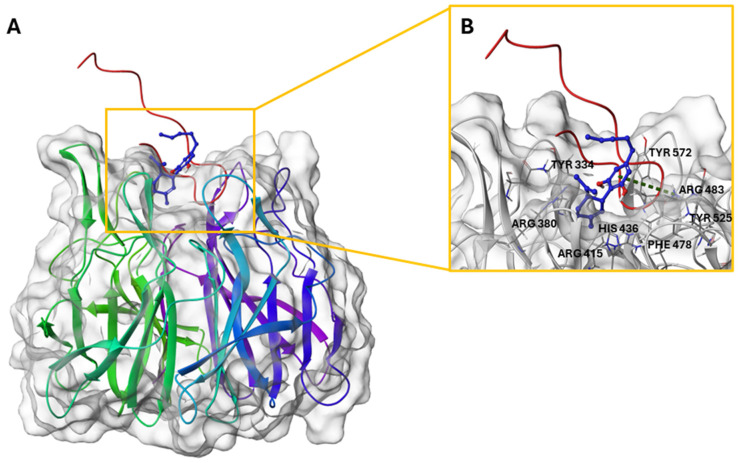
(**A**) Three-dimensional structure of Keap1-Nrf2 peptide (red loop) in complex with CBD. (**B**) Binding mode of CBD (colored by atom type: C blue, O red, polar H white). π-cation interaction is reported in green.

Therefore, molecular docking calculations were carried out employing the human Keap1 Kelch domain bound to the 16mer Nrf2 peptide (PDB: 2FLU) [70]. Analysis and visual inspection of docking poses revealed that CBD is able to interact with Keap1 in the proximity of the Nrf2 binding site. In particular, CBD establishes a π-cation interaction with Arg483 [68], a key residue in the binding between Keap1 and Nrf2 (Figure 6), and it is situated near several hydrophobic residues within the Keap1 binding pocket, e.g., Phe478 and Gly480. These spatial connections suggest the presence of stabilizing hydrophobic contacts that may contribute to the overall binding affinity of CBD. Furthermore, hydrogen bonds are predicted between the hydroxyl groups of CBD and polar residues such as His436, as reported in Figure S8, indicating a potential alternative binding of CBD to the protein counterpart. Collectively, these interactions support the hypothesis that CBD can engage Keap1 through a combination of π-cationic, hydrophobic, and hydrogen bonding interactions, potentially influencing its interaction with Nrf2. These interactions suggest that CBD may act as a weak binder of Keap1, potentially influencing the regulation of the Nrf2 pathway without drastically interfering with its mechanism of action.

Although further research is needed to establish the mechanism and potential value of the effects described here better, CBD represents a significant addition to the rich repertoire of bioactivities ascribed to this phytocannabinoid.

## 3. Materials and Methods

### 3.1. Standards and Reagents

DPPH• (1,1-Diphenyl-2-picrylhydrazyl), ascorbic acid, Folin-Ciocalteu reagent, gallic acid, sodium carbonate, 2,4,6-tri(2-pyridyl)-s-triazine (TPTZ), 6-hydroxy-2,5,7,8-tetramethlchroman-2-carboxylic acid (Trolox), and 3,3′,4′,5,7-Pentahydroxyflavone dehydrate (Quercetin) were obtained from Sigma Chemical Co. (St. Louis, MO, USA). All solvents used for extraction were purchased from VWR Intl. (West Chester, PA, USA). Acetonitrile, methanol, and water of LC-MS grade were used for LC-ESI-HR-MS analysis (Ultra LC ROMIL Pure Chemistry, Cambridge, UK). For H1-NMR analysis, chloroform and methanol-d (deuterated) were purchased from Sigma Chemical Co. (St. Louis, MO, USA). All other chemicals were of analytical grade.

### 3.2. Plant Material

The dried biomass of *C. sativa* Kompolti variety was kindly provided in 2023 by the Italian agricultural cooperative society, MarcheSana (CANNAPA^®^) (Via di Villa Giulia, Loc. S. Biagio, Fano, Pesaro Urbino, Italy) and certified with the content of Δ9-THC below 0.2% (*w*/*w*). [Batch number seeds: B31270201900001]. Kompolti is an EU-certified seed dioecious hybrid that is currently widespread throughout Europe and chosen for consumption due to the superior quality of inflorescences with a low THC content (≤0.2%) and high CBD percentages (2–10%). As a result, Kompolti is an excellent raw material for producing inflorescences and biomass for extraction (CBD) for food and therapeutic purposes. Hemp inflorescences were manually separated from seeds, and then the samples were stored at +4.0 °C until chemical analysis was required. A voucher specimen is deposited under No. CANK-48-2022 in the Herbarium of the University of Molise (Pesche, Isernia, Italy).

### 3.3. Water Infusion (WI) Preparation of Kompolti Variety

Dried inflorescences (14.0 g) were crushed and added to 200 mL of boiling distilled water in a glass beaker and left to stand at room temperature for 15 min to simulate the possible home-made use (decoction, infusion) of hemp inflorescences. The mixture was then filtered and concentrated to dryness under reduced pressure using a rotary evaporator at 40°C to yield 88.3 mg of aqueous extract. The dry residue (WI) was submitted to Kupchan partitioning procedure [37] to yield four extracts: *n*-hexane (2.3 mg), CHCl_3_ (48.0 mg), *n*-BuOH (127 mg), and water *residual* extracts (1.63 g), each extract was submitted to ^1^H NMR experiments. The chloroform extract was purified on a semi-preparative Nucleodur 100-5 C18 column (10 μm, 250 mm × 4.6 mm i.d) (flow rate 4.0 mL/min) using MeOH/H_2_O (8:2) as eluent to obtain pure CBDA (1.3 mg t_R_ = 22.4 min) and CBD (4.7 mg t_R_ = 28.0 min).

### 3.4. Sample Preparation and NMR Analyses

NMR experiments were acquired on a Bruker DRX-600 spectrometer (Bruker Bio-SpinGmBH, Rheinstetten, Germany) at 600 MHz. The acquisition parameters [61] were: FID size = 64 K, spectral width = 15.00 ppm, receiver gain = 90.5, scans = 512, and number of dummy scans = 4. Data acquisition for the polar extract was also achieved by using a zg pulse sequence (Bruker 1D). The analyses were based on 1D ^1^H-NMR

Dried and lyophilized polar extracts were dissolved in CD_3_OD, referred to CHD_2_OD (δ_H_ 3.31 and δ_C_ 49.0), and apolar extracts were solubilized in CDCl_3_ (δ_H_ 7.26, δ_C_ 77.0). The ^1^H NMR assignments of CBD and CBDA signals are described in Supplementary Materials and reported in Figures S6 and S7, respectively.

### 3.5. Metabolites Identification in the Different Extracts of Kompolti Hemp Variety by LC-ESI-Q-Exactive MS/MS Experiments

The CHCl_3_ and n-hexane extracts were dissolved in methanol of LC-MS grade (Romil), while n-butanol and water residue extracts were dissolved in H_2_O of LC-MS grade (Romil) and sonicated. After centrifugation (5000 rpm, 15 min, room temperature), the samples were placed into micro-vials with a final concentration of 1 mg/mL following centrifugation (5000 rpm, 15 min, room temperature).

All samples were analyzed using a LC system (Ultimate 3000) coupled to an Orbitrap Q-Exactive Classic mass spectrometer (ThermoFisher Scientific, Bremen, Germany). Liquid chromatography (LC) was performed with a Luna Omega C18 LC Column (3 µm, 150 × 2.1 mm) (Phenomenex, Torrance, CA, USA) for separation. A 5 µL full loop injection was utilized for the water and butanol extracts, and a gradient program was used for 30 min, starting at 5% and working up to 95% of the B phase. Phase A consisted of H_2_O with 0.1% formic acid, while Phase B comprised CH_3_CN with 0.1% formic acid. A 5 µL full loop injection was utilized for CHCl_3_ and hexane extracts, and a gradient program was employed for 30 min, ranging from 30% to 95% of the B phase. The system included an electrospray ionization source that operated in both positive and negative ion switching modes. Full ion MS was set for each extract, and all ion fragmentation (Data-Dependent Scan) was set as scan events for each extract, with the MS/MS fragmentation of the first five most intense ions in the full scan. Operation parameters both for negative and positive ion mode were as follows: FTMS scan mode with a mass range from 150 to 1500 *m*/*z* with a resolution of 70,000; spray voltage 3000; capillary temperature 275 °C; sheath and auxiliary gas flow (N_2_), 40 and 5; sweep gas 0; spray voltage 5.

### 3.6. Determination of Total Phenolic Content and Flavonoid Content

#### 3.6.1. Extraction Procedure (HEC)

A 0.45 g sample of *Cannabis* inflorescences was extracted in a capped centrifuge tube with 9 mL of methanol (80% *v*/*v*) [51,52]. The samples were then sonicated in an ultrasonic bath (Sonica, Ultrasonic cleaner, Milan, Italy) for 45 min, followed by incubation at room temperature (25 °C) in the dark for 15 min. Afterward, the samples were centrifuged for 15 min at 5000 rpm. The supernatant was carefully transferred into new tubes, and the extracts were stored at 4 °C in the dark until further analysis.

#### 3.6.2. Folin-Ciocalteu Assay

The total phenolic content was measured using the Folin–Ciocalteu method, as outlined by Singleton et al. [71]. To 0.5 mL of the appropriately diluted sample extract, 0.5 mL of Folin-Ciocalteu reagent (diluted 1:10 *v/v* with water) was added. The mixture was allowed to stand for 5 min, followed by the addition of 3 mL of Na_2_CO_3_ (7%; *w*/*v*). The final volume was adjusted to 4.5 mL with water. A blue colour developed, and after 1 h, the absorbance was measured at 765 nm using a spectrophotometer (Shimadzu UV-1601, Kyoto, Japan), with the blank consisting of all reagents and solvents, excluding the test compounds. The total phenolic content was expressed as gallic acid equivalents (mg g^−1^ GAE) based on a calibration curve (1–10 μg mL^−1^) of gallic acid. All experiments were replicated three times, and the results are expressed as mean values.

#### 3.6.3. Total Flavonoid Content (TFC)

The total flavonoid content in the extracts was determined using the method described by Zhishen [72]. Briefly, a 100 μL aliquot of the *cannabis* extract, undiluted, was mixed with 1.3 mL of distilled water, followed by the addition of a 5% NaNO_2_ solution (80 μL). After 6 min, 150 μL of a 10% AlCl_3_ × 6 H_2_O solution was added and allowed to stand for another 5 min in the dark before adding 500 μL of a 1.0 M NaOH solution. The mixture was then brought to a final volume of 3.5 mL with distilled water and mixed thoroughly. The absorbance was immediately measured against the blank (the same mixture without the sample) at 510 nm (Shimadzu UV-1601, Kyoto, Japan). The results were calculated and expressed as mg of quercetin equivalents (mg g^−1^ QUE) using the quercetin calibration curve. The linearity range of the calibration curve was from 2 to 100 μg mL^−1^ for quercetin. All experiments were performed in triplicate, and the results are presented as mean values.

### 3.7. Antioxidant Capacity

The antioxidant capacity was determined using two different methods: DPPH^•^ (1,1-Diphenyl-2-picrylhydrazyl) radical scavenging activity and FRAP (Ferric-Reducing Antioxidant Power) procedures. Hydroalcoholic extract (HEC) and water infusion (WI), prepared as described in Section 3.6.1 and Section 3.3, respectively, were used to evaluate antioxidant activity.

#### 3.7.1. DPPH Radical Scavenging Activity

The free radical scavenging capacity of extracts was evaluated by DPPH assay according to the procedure reported by Lopez et al. [73] and slightly modified.

An amount of 100 μL of the sample solution, suitably diluted, was added to 500 μL of a freshly prepared DPPH^•^ methanolic solution (27 μg mL^−1^, diluted until reaching an absorbance in the range 0.8 and 0.9) and 2 mL of methanol (80% *v*/*v*) for Hydroalcoholic Extract of *C. sativa* (HEC). For the Water Infusion (WI) extract, instead of methanol, 2 mL of water was added to avoid interfering with the extraction process. The reaction mixture was kept in the dark for 30 min; when the DPPH radical reacts with an antioxidant compound, it is reduced and changes colour, leading to a color change from purple to yellow. The colour changes were read as absorbance at 517 nm (Shimadzu UV-1601 spectrophotometer) using methanol (80% *v*/*v*) as a blank. The antioxidant activity is expressed as mg of Trolox Equivalent (TE) per kilogram (mg kg^−1^ of TE). Trolox, a water-soluble analog of vitamin E, is used as a standard reference compound for antioxidant activity. To determine the Trolox Equivalent, the antioxidant activity of the sample extract was compared to that of various concentrations of Trolox, and a calibration curve was generated. The concentration of Trolox (mg mL^−1^) corresponding to the same antioxidant capacity as the sample was calculated, and the result was expressed in terms of mg of Trolox Equivalent per kilogram of the sample.

#### 3.7.2. FRAP Assay

The FRAP assay was performed as previously described by Benzie et al. [74], with modifications. The working FRAP reagent was prepared by mixing acetate buffer (300 mM, pH 3.6) with TPTZ (2,4,6-tripyridyl-s triazine) solution (10 mM in 40 mM HCl) and with FeCl_3_ × 6 H_2_O (20 mM) in a ratio 10:1:1. A total of 100 μL of each extract (HEC and WI), suitably diluted, was reacted with 3.0 mL of FRAP shaken vigorously and incubated at 37 °C for 30 min. The absorbance was measured at 593 nm (Shimadzu UV-1601 spectrophotometer). The in vitro antioxidant activity quantification was performed using a standard Trolox curve in a concentration range between 0.1 to 6 μg mL^−1^. The results were expressed in mmol of Trolox equivalents per kilogram of dry weight (mmol TE Kg^−1^ d.w.).

### 3.8. Macro and Microelements Content Determination

A sample pretreatment based on an acid digestion procedure was required for element analysis, performed using closed-vessel microwave digestion equipment (ETHOS EASY, Milestone, Sorisole, Italy), as described by Menezes et al. [75], with some modifications. Approximately 0.3 g of sample was accurately weighed into Teflon vessels, to which 4 mL of concentrated nitric acid (HNO_3_, TraceSELECT Ultra for ultratrace analysis, 67–69%, Honeywell Fluka), 2 mL of hydrogen peroxide (H_2_O_2_, for trace analysis 30%, Merck), and 4 mL of ultrapure water were added. The digestion program was carried out under the following operating conditions: 5 min at a temperature ranging from 0 to 180 °C (step 1), followed by 5 min at a constant temperature of 180 °C (step 2), with a constant microwave power of 1800 W. After digestion, the contents were filtered using 0.45 μm PTFE filters, transferred to a 25.0 mL volumetric flask, and diluted to volume with ultrapure water. As for the infusion (WI), the samples were acidified to a pH < 2 to ensure complete solubilization of the metals, prevent the formation of precipitates, and maintain optimal ionization in the ICP-OES plasma. The samples were then analysed using an inductively coupled plasma optical emission spectrometer (ICP-OES) (5800 ICP-OES, Agilent, Santa Clara, CA, USA). Argon (purity > 99.995%) was employed as the plasma and carrier gas. Calibration was performed using a standard solution of 100 mg mL^−1^ of calcium, iron, potassium, phosphorus, and sulfur, and a standard solution of 10 mg/mL of copper, magnesium, manganese, and nickel, both dissolved in 10% nitric acid (HNO_3_), supplied by Supelco (Multielement standard solution 5 for ICP and Metalloid and non-metal mix for ICP). The mineral contents of each sample were analyzed in triplicate (*n* = 3).

### 3.9. Determination of Biological Activity

#### 3.9.1. Cell Culture

SH-SY5Y human neuroblastoma cells were cultured at 5% CO_2_ in DMEM medium at high glucose (D6429, Merck Life Science, Milan, Italy), containing 10% (*v*/*v*) foetal bovine serum (FBS, F7524, Merck Life Science, Milan, Italy) and 1% penicillin/streptomycin solution (P06-07100, PAN Biotech, Aidenbach, Germany). For the experiments, 250,000 SH-SY5Y cells were seeded. After 5 h, cell differentiation was induced in DMEM containing 1% FBS and retinoic acid 10 μM (R2625, Merk Life Science, Milan, Italy) for 72 h. Differentiated cells were then pre-treated with CBD (1 μM) or vehicle (DMSO, dilution 1:1000 in cell culture media). To facilitate solubilization in the growth medium, CBD extracted from *Cannabis sativa* L. plant was first dissolved in DMSO (at a final concentration of 100 μM), before being added to DMEM. After 24 h, cells were exposed to rotenone (0.1 μM, R8875, Merck Life Science, Milan, Italy) in the presence or absence of CBD for the following 48 h for cell count, and 24 h for immunofluorescence experiments.

#### 3.9.2. Immunofluorescence

SH-SY5Y cells were plated on sterilized coverslips coated with poly-L-lysine. After pharmacological treatments, cells were fixed in a 4% paraformaldehyde (D1408, Merck Life Science, Milan, Italy) solution in DPBS (D1408, Merck Life Science, Milan, Italy) at pH 7.4 for 10 min at room temperature. 0.1% Triton X-100 (Merck Life Science, Milan, Italy) solution was used for cell permeabilization for 5 min. Subsequently, samples were blocked in 3% bovine serum albumin (BSA, A3912, Merck Life Science, Milan, Italy) in DPBS with 0.1% Triton X-100 for 1 h at room temperature. Cells were then incubated overnight with primary antibodies: 8-OHdG (sc-66036, Santa Cruz Biotechnology, Dallas, TX, USA; dilution 1:100), Nrf2 (sc-365949, Santa Cruz Biotechnology, Dallas, TX, USA; dilution 1:100), PPARα (sc-398394, Santa Cruz Biotechnology, Dallas, TX, USA; dilution 1:100), PPARγ (Ab59256, Abcam, Cambridge, UK; dilution 1:100). Subsequently, cells were incubated for 1 h with anti-mouse secondary antibody Alexa Fluor 555 (A28180, Thermo Fisher Scientific, Waltham, MA, USA; dilution 1:300) or anti-rabbit secondary antibody Alexa Fluor 488 (A27034, Thermo Fisher Scientific, Waltham, MA, USA; dilution 1:300). DAPI (D9542, Merck Life Science, Milan, Italy) was used to counterstain cell nuclei. Finally, coverslips were mounted with Fluoroshield mounting medium (F6182, Merck Life Science, Milan, Italy). Samples were then examined using confocal microscopy (TCS SP8, Leica, Wetzlar, Germany) at 40× magnification, and images were acquired with LAS X software (version 3.5.5) (Leica Camera, Wetzlar, Germany) for Windows 10. Acquisition parameters, including laser intensity, photomultiplier gain, and image magnification, were kept unchanged across all experimental groups. Quantification of protein levels was performed by calculating the mean fluorescence intensity per cell area using ImageJ software v.154d (National Institutes of Health, Bethesda, MD, USA) for Windows 11.

### 3.10. Molecular Docking

The crystallographic structure of Keap1 in complex with the Nrf2 peptide, retrieved from the Protein Data Bank (PDB code: 2FLU) [70], was used for docking calculations. The complex was prepared using the Protein Preparation Wizard (Schrödinger suite) [76,77] at a pH of 7.4 ± 1.0, removing water molecules, adding missing hydrogen atoms, and assigning bond orders. The docking grid used for all calculations featured innerbox dimensions of 10 Å, and outerbox dimensions of 20 Å. The center grid coordinates were set as 6.84 Å (x), 13.13 Å (y), and 3.25 Å (y) to cover the binding site region.

The 2D structure of cannabidiol was drawn using the Maestro 2D sketcher tool [78] in the Schrödinger Suite and subsequently prepared using LigPrep software (version 5.7) [79], accounting for the protonation state at a pH = 7.4 ± 1.0 and minimizing the structure with OPLS 2005 force field, retaining the specified chirality and generating all the possible tautomers.

All molecular docking experiments were performed using Glide [80,81,82,83] software and setting the Standard Precision (SP) mode. From the 10,000 poses generated in the initial docking phase, 800 conformations were retained and evaluated, which were subjected to the minimization step with an energy threshold of 0.15 kcal/mol. Finally, a maximum of 20 poses were saved for subsequent qualitative analysis through visual inspection.

### 3.11. Statistical Analysis

Data were expressed as mean ± SD (standard deviation). Statistical significance was assessed using the one-way analysis of variance (ANOVA) test, followed by Tukey’s post hoc tests. *p* value < 0.05 was considered to indicate a statistically significant difference. Statistical analysis and graph editing were carried out using GraphPad Prism 8.4.2 (GraphPad, La Jolla, CA, USA) for Windows 11.

## 4. Conclusions

The hot water extraction used in this study is a method to simulate the production process of *Cannabis* beverages. The WI of inflorescences of the industrial hemp *Kompolti* cultivar is a source of phytochemicals that have received increased attention for their human health benefits. Several of these activities are mainly related to non-nutritional compounds such as polyphenols, flavonoids, and non-psychoactive cannabinoids. Thanks to the application of LC-MS/MS analysis, the results of the four extracts (*n*-hexane, CHCl_3_, *n*-BuOH, and water residual extract) obtained by a modified Kupchan’s partitioning procedure of the WI of *C*. *sativa* inflorescence was well defined, highlighting the presence of phytocannabinoids, especially CBD and CBDA, in *n*-hexane and CHCl_3_ fractions, while the presence of flavonoids and phenolic acid in n-BuOH and water fractions was evidenced. This result demonstrates that the infusion of *Kompolti* has its own chemical profile, responsible for the nutritional and sensory properties linked to its components, which could be used as ingredients for health products, functional foods, dietary supplements, and cosmetics.

The inflorescence extracts WI also showed good mineral content, with Ca, K, Mg, and P comprising the main elements detected, while Fe, Cu, Mn, and Ni were highlighted as micronutrients. CBD, the main cannabinoid component, has been isolated and evaluated in a cellular model of PD. Morphological evaluations and cell counts highlighted that CBD protects against rotenone-induced cell death without recovering neuronal morphology. Interestingly, immunofluorescence analysis showed a significant reduction in the expression of the oxidative stress marker 8OH(d)G; furthermore, CBD increased the immunopositivity of the main transcription factors involved in the antioxidant cytoprotective response.

Since the therapeutic potential of CBD has been linked to the Nrf2 signalling pathway, which is involved in the regulation of ROS metabolism in several physiopathological conditions, in this study, molecular docking has been carried out to gain a clearer understanding of the potential interaction of CBD with Nrf2 pathway. Analysis and visual inspection of docking poses revealed that CBD could be capable of interacting with Keap1 in the vicinity of the Nrf2 binding site, thus rationalizing, at the molecular level, its observed biological effects, influencing the Nrf2 pathway’s control without significantly altering its mode of action.

Therefore, although CBD showed a notable antioxidant capacity, promoting SH-SY5Y viability after rotenone toxic insult, further investigation is required to better understand other regulatory events, such as the functional activity of the key enzymes involved in the antioxidant response. In summary, this multidisciplinary approach has provided further insight into the human health properties of *C. sativa* L. *Kompolti* infusions. When consumed as a beverage as part of a normal diet, the phytoconstituents could provide health benefits through their antioxidant activity in various diseases and would be promising for protection against environmental stresses that contribute to inflammatory processes, cancer, and other degenerative diseases.

## Figures and Tables

**Table 1:** Metabolites identified in the *n*-hexane extract by LC-MS and MS/MS analysis with Compound Discoverer software version 3.3.3 and research in literature.

Positive Ion Mode					
Name	Formula	ppm	[M + H]^+^	Rt	MS/MS
(−)-Caryophyllene oxide	C_15_H_24_O	−0.7	203.1793	14.2	147.1166/133.1012/121.1011/105.0701/95.0858
Unsaturated cannabispirol	C_15_H_18_O_3_	−1.4	247.1325	15.08	211.1113/169.1008/183.1165
11-nor-9-carboxy-Δ9-tetrahydrocannabinol	C_21_H_28_O_4_	−0.6	345.2058	19.2	327.1587/285.1121/205.0488
Δ9-THCA derivative (C4)	C_21_H_28_O_4_	−0.8	345.2057	22.9	327.1592/193.1218/205.0858
Cannabidiolic acid (CBDA)	C_22_H_30_O_4_	−3.1	359.2206	24.1	219.1012/261.1479/285.1484
Cannabidiol (CBD)	C_21_H_30_O_2_	−1.5	315.2312	24.8	259.1689/193.1221/135.1168/181.1219
Octadecanamide	C_18_H_37_NO	−1.6	284.2943	34.2	141.1274
**Negative Ion Mode**					
**Name**	**Formula**	**ppm**	**[M** − **H]^−^**	**Rt**	**MS/MS**
9-OxoODE	C_18_H_30_O_3_	−1.3	295.2264	22.4	277.2168/195.1383

**Table 2:** Metabolites identified in the CHCl_3_ extract by LC-MS and MS/MS analysis with Compound Discoverer software version 3.3.3 and research in the literature.

Positive Ion Mode					
Name	Formula	ppm	[M + H]^+^	Rt	MS/MS
Hedione	C_13_H_22_O_3_	−0.88	209.1534	5.22	125.0960/153.0908
5-(1-Butyl-1-hydroxypentyl)-1.3-benzenediol	C_15_H_24_O_3_	−1.9	253.1793	5.55	217.1583/159.1166
2,6-Dimethoxy-4-propylphenol	C_11_H_16_O_3_	−3.04	197.1168	6.95	176.1064/161.0960/135.1168
5-(1-Butyl-1-penten-1-yl)-1,3-benzenediol	C_15_H_22_O_2_	−0.55	235.1691	7.75	186.1852
β-cannabispiranol	C_15_H_20_O_3_	−0.2	249.1483	9.05	231.1375/213.1871/189.1272
Diosmetin	C_16_H_12_O_6_	−0.56	301.0705	12.34	286.0469/258.0522
α-cannabispiranol	C_15_H_20_O_3_	0.8	249.1482	13.51	231.1387/189.0909/163.0751
Humulene	C_15_H_24_	−0.57	205.195	14.14	121.0648
6-Gingerol	C_17_H_26_O_4_	−0.81	277.1796	15.49	304.6685/310.8908
Cannabielsoic acid A	C_22_H_30_O_5_	1.3	375.2152	17.0	357.2057/339.1952/275.1275
Cannabicoumarononic acid A	C_22_H_28_O_5_	−0.61	373.2007	17.83	355.1899/337.1791/331.1793
Cannabidiolic acid (CBDA)	C_22_H_30_O_4_	−0.27	359.2204	19.65	341.2105/261.1480/219.1014
Epoxyhexahydrocannabinol acetate	C_23_H_32_O_4_	−0.90	373.2370	21.80	355.1899/337.1791/331.1793
Δ9-THCA derivative (C1)	C_18_H_23_O_4_	−1.8	303.1585	19.8	285.1479/205.0857/163.0387
Cannabichromenic acid (CBCA)	C_22_H_30_O_4_	−0.31	359.2206	24.3	341.2105/261.1480/219.1014
Cannabidiol (CBD)	C_21_H_30_O_2_	−1.41	315.2314	25.1	259.1688/193.1222
Palmitic amide	C_16_H_33_NO	−2.03	256.263	29.8	88.0761
**Negative Ion Mode**					
**Name**	**Formula**	**ppm**	**[M − H]^−^**	**Rt**	**MS/MS**
15,16-DiHODE	C_18_H_32_O_4_	−1.09	311.2224	16.3	201.1124/223.1697
6,7-Epoxy-CBGA	C_22_H_30_O_5_	1.6	375.2172	19.6	357.1796/331.1902/313.2167/261.1493/203.1069
11-Hydroxy-Δ9-THC	C_21_H_30_O_3_	−0.27	329.2121	20.2	311.2017/271.1700
Cannabielsoic acid A	C_22_H_30_O_5_	1.3	373.2017	22.6	357.2057/339.1952/275.1275
16-Hydroxyhexadecanoic acid	C_16_H_32_O_3_	0.02	271.2279	27.0	225.2219/253.2170

**Table 3:** Metabolites identified in the *n*-BuOH extract by LC-MS and MS/MS analysis with Compound Discoverer software version 3.3.3 and research in the literature.

Positive Ion Mode					
Name	Formula	(ppm)	[M + H]^+^	Rt	MS/MS
Phenylalanylthreonine	C_13_H_18_N_2_O_4_	−1.2	267.1336	2.88	121.0648
D-(+)-Tryptophan	C_11_H_12_N_2_O_2_	−0.6	205.097	4.91	146.0599/188.0704
L-Phenylalanine	C_9_H_11_NO_2_	0.02	166.0863	6.11	105.0337/149.0598
Kaempferol 3-O-rutinoside	C_27_H_30_O_15_	−1.0	595.165	11.3	329.0651/287.0548/433.1829
Orientin	C_21_H_20_O_11_	−2.5	449.1067	11.62	287.0923
Apigenin 7-*O*-rutinoside	C_27_H_30_O_14_	−2.8	579.1691	11.9	433.1125/271.0605/313.0703/397.0918
Vitexin	C_21_H_20_O_10_	−0.5	433.1127	12.2	313.0703/397.0914/271.0612
Luteolin 7-*O*-glucuronide	C_21_H_18_O_12_	−0.3	463.087	12.7	287.0545
Apigenin 7-glucuronide	C_21_H_18_O_11_	−2.7	447.0909	13.72	271.0596
Hispidulin 7-glucuronide	C_22_H_20_O_12_	−0.3	477.1026	13.95	301.0703
αlpha-Cannabispiranol	C_15_H_20_O_3_	−1.9	249.1480	16.14	231.1375/163.0750/137.0596
Luteolin	C_15_H_10_O_6_	−0.8	287.0548	16.4	153.0182/173.1322
Andrographolide	C_20_H_30_O_5_		351.214	17.1	281.0828/221.0796
Apigenin	C_15_H_10_O_5_	−0.61	271.0599	17.83	153.0186
Cannabidiolic acid (CBDA)	C_22_H_30_O_4_	−0.36	359.2216	22.86	261.1482
(9Z)-2-Oxo-9-octadecenamide	C_18_H_33_NO_2_	−1.59	296.2579	26.02	279.2316/261.2209/233.2260
Cannabichromenic acid (CBCA)	C_22_H_30_O_4_	−2.3	359.2216	27.318	219.1014/261.1482
Tetrahydrocannabinol	C_21_H_30_O_2_	−2.28	315.2311	27.87	259.1689/193.1222
Palmitic amide	C_16_H_33_NO	−2.38	256.2629	32.17	
Octadeca-9,12-dienal	C_18_H_32_O	−1.13	265.2523	32.57	207.1236

In this extract, the same metabolites were detected in both negative and positive ion modes, but for simplicity, only the positive ion mode is reported.

**Table 4:** Metabolites identified in the H_2_O extract by LC-MS and MS/MS analysis with Compound Discoverer software version 3.3.3 and research in the literature.

Positive Ion Mode					
Name	Formula	ppm	[M + H]^+^	Rt	MS/MS
N6,N6,N6-Trimethyl-L-lysine	C_9_H_20_N_2_O_2_	0.3	189.1598	1.69	
L-(+)-Arginine	C_6_H_14_N_4_O_2_	−1.5	175.1187	1.75	146.9600/118.9649
N-(1-Deoxy-1-fructosyl)proline	C_11_H_19_NO_7_	−0.8	278.1232	2.28	260.1125/242.1017/116.0707
Citric acid	C_6_H_8_O_7_	−1.2	210.0606	2.65	
Leucylproline	C_11_H_20_N_2_O_3_	0.3	229.1548	3.94	
Guanosine	C_10_H_13_N_5_O_5_	−0.05	284.099	3.97	152.0565
Kaempferol 3-galactoside-7-rhamnoside	C_27_H_30_O_15_	−0.06	595.1657	11.60	287.0544
Apigenin 7-O-rutinoside	C_27_H_32_O_15_	−0.11	579.1706	11.84	433.1125/313.0702/271.0602
Luteolin glucuronide	C_21_H_18_O_12_	−0.41	463.0869	12.74	287.0547
Apigenin 7-O-glucuronide	C_21_H_18_O_11_	−3.16	447.0919	13.59	271.0596
Octadeca-9,12-dienal	C_18_H_32_O	−1.93	265.2520	32.65	207.1238
**Negative Ion Mode**					
**Name**	**Formula**	**(ppm)**	**[M − H]^−^**	**Rt** **(min)**	**MS/MS**
Galactonic acid	C_6_H_12_O_7_	−4.28	195.0502	1.81	129.0180/75.0074
Galactaric acid	C_6_H_10_O_8_	−3.21	209.0296	1.82	
L-Gulonolactone	C_6_H_10_O_6_	−3.25	223.0454	1.85	
Citric acid	C_6_H_8_O_7_	−4.75	191.0188	2.07	
Guanosine	C_10_H_13_N_5_O_5_	−0.36	282.0843	3.98	150.0409
Protocatechuic acid hexoside	C_13_H_16_O_9_	0.64	315.0724	6.76	153.0183/109.0283
Tuberonic acid glucoside	C_18_H_28_O_9_	3.3	387.1662	10.16	207.1018/163.1117
Sinapoylglucose	C_17_H_22_O_10_	−4.19	385.1137	10.33	223.0605/208.0381/179.0703
Apigenin 7-*O*-rutinoside	C_27_H_30_O_14_	−1.74	577.1563	11.84	269.0453/413.0869
Coutaric acid	C_13_H_12_O_8_	−1.69	295.0454	12.80	173.0082/111.0074/85.0281
11-hydroxy THCA-2-glucuronic acid	C_28_H_38_O_11_	1.76	549.234	13.91	343.1913/189.0923

**Table 5:** Total Polyphenol and Flavonoid content of Hydroalcoholic Extract of C. sativa (HEC) and Water Infusion (WI). Total antioxidant capacity determined by DPPH and FRAP assays.

Sample	Total Phenolic	Flavonoids	DPPH	FRAP
	*(mg g^−1^ GAE d.w.)*	*(mg g^−1^ QUE d.w.)*	*(mg Kg^−1^ TE d.w.)*	*(m* *mol Kg^−1^ TE d.w.)*
**HEC**	3.29 ± 0.22	1.39 ± 0.3	58.87 ± 0.54	2.76 ± 0.03
**WI**	1.00 ± 0.13	0.48 ± 0.1	11.28 ± 0.04	1.49 ± 0.01

GAE: Gallic acid equivalents. QUE: Quercetin equivalents. TE: Trolox equivalents. Each value is a mean ± SD of triplicate analysis.

**Table 6:** Microelements and macroelements quantified by ICP-OES in mineralised *Cannabis* inflorescences (MCI) and water infusion (WI).

		Inflorescence MCI	Infusion WI
Microelements *(mg Kg^−1^)*	Wavelength *(nm)*	*Mean ± SD*	
Cu	327.395	7.99 ± 0.59	3.43 ± 0.21
Fe	238.204	365.39 ± 37.03	2.86 ± 0.14
Ni	231.604	1.12 ± 0.29	0.57 ± 0.04
Ba	455.403	33.94 ± 1.12	1.00 ± 0.07
Co	230.786	0.24 ± 0.07	<0.07
Cr	267.716	0.79 ± 0.11	0.02 ± 0.01
Pb	220.353	1.43 ± 0.26	<0.14
V	292.401	0.61 ± 0.12	<0.14
Zn	213.857	58.41 ± 0.82	19.00 ± 0.11
B	249.772	66.91 ± 1.96	8.57 ± 0.08
**Macroelements** *(**g Kg^−1^**)*			
Ca	393.366	50.40 ± 0.24	5.34 ± 0.09
K	766.491	12.65 ± 0.38	11.16 ± 0.17
Mg	279.553	2.92 ± 0.12	1.17 ± 0.08
P	214.914	2.77 ± 0.16	0.53 ± 0.07
S	181.972	1.71 ± 0.09	0.72 ± 0.01
Si	251.611	2.20 ± 0.11	0.27 ± 0.02

Mean ± SD (standard deviation), *n* = 3.

## Data Availability

Data are contained within the article and Supplementary Materials.

## Supplementary Materials

The following supporting information can be downloaded at: https://www.mdpi.com/article/10.3390/plants14101473/s1. Figure S1. ^1^H NMR (CDCl_3_, 600 MHz) of *n*-hexane extract obtained from the modified Kupchan partitioning procedure of the hot water infusion (WI) of the inflorescences of *Cannabis sativa* L. *Kompolti* variety. Some selected ^1^H NMR signals associated with CBD and CBDA have been evidenced. Figure S2. ^1^H NMR (CDCl_3_, 600 MHz) of CHCl_3_ extract obtained from the modified Kupchan partitioning procedure of the hot water infusion (WI) of the inflorescences of *Cannabis sativa* L. *Kompolti* variety. Some selected ^1^H NMR signals associated with CBD and CBDA have been evidenced. Figure S3. ^1^H NMR (CD_3_OD, 600 MHz) of *n*-BuOH extract (full (panel A) and enlarged (from 9.2 to 5.4 ppm) spectrum region (panel B)) obtained from the modified Kupchan partitioning procedure of the hot water infusion (WI) of the inflorescences of *Cannabis sativa* L. *Kompolti* variety. Figure S4. ^1^H NMR (CD_3_OD, 600 MHz) of water extract (1D sequence without (panel A) and with (panel B) f1 presaturation and spectrum enlarged (from 9.2 to 5.0 ppm) region (panel C)) obtained from the modified Kupchan partitioning procedure of the hot water infusion (WI) of the inflorescences of *Cannabis sativa* L. *Kompolti* variety. Figure S5. Chemical structures of Cannabidiol (CBD) and Cannabidiolic acid (CBDA). Figure S6. 1H NMR (CD3OD, 400 MHz) of Cannabidiol (CBD) and ^1^H NMR assignments of CBD in CD_3_OD. Figure S7. 1H NMR (CD3OD, 400 MHz) of Cannabidiolic acid (CBDA) and ^1^H NMR assignments of CBDA in CD_3_OD. Figure S8. Alternative binding mode of CBD (colored by atom type: C blue, O red, polar H white) in complex with the three-dimensional structure of Keap1-Nrf2 peptide complex (red loop). H bond is reported in yellow. References
