# Supplementary material for: Chemical Profiling of Polyphenolic Fraction of Cannabis sativa L. vr. Kompolti Industrial Inflorescences: Insights into Cannabidiol Neuroprotective Effects in a Cellular Model of Parkinson’s Disease
Source: Plants (Basel). 2025 May 14;14(10):1473. doi: 10.3390/plants14101473 (PMC12115228; doi:10.3390/plants14101473)
Supplement: Supplementary file 1 [file plants-14-01473-s001.zip › plants-3598086-supplementary.pdf]

Supporting Information

Chemical Profiling of Polyphenolic Fraction of *Cannabis sativa* L. vr. *Kompolti* Industrial Inflorescences: Insights into Cannabidiol Neuroprotective Effects in a Cellular Model of Parkinson's Disease

Francesca Fantasma [1,†], Gilda D'Urso [2,†], Noemi Martella [1,†], Alessandra Capuano [2], Eleonora Boccia [2], Vadym Samukha [1], Vincenzo De Felice [1], Gabriella Saviano [1], Federico Trombetta [3], Gianluigi Lauro [2], Marco Segatto [1], Maria Giovanna Chini [1,*], Giuseppe Bifulco [2], Agostino Casapullo [2,*] and Maria Iorizzi [1]

[1] Department of Biosciences and Territory, University of Molise, Contrada Fonte Lappone, 86090 Pesche, Italy; fantasma@unimol.it (F.F.); noemi.martella@unimol.it (N.M.); v.samukha@studenti.unimol.it (V.S.); defelice@unimol.it (V.D.F.); saviano@unimol.it (G.S.); marco.segatto@unimol.it (M.S.); iorizzi@unimol.it (M.I.)

[2] Department of Pharmacy, University of Salerno, Via Giovanni Paolo II 132, 84084 Fisciano, Italy; gidurso@unisa.it (G.D.); acapuano@unisa.it (Al.C.); eboccia@unisa.it (E.B.); glauro@unisa.it (G.L.); bifulco@unisa.it (G.B.)

[3] Societa Cooperativa Agricola MarcheSana, Localita San Biagio 40, 61032 Fano, Italy; federico.trombetta@gmail.com

* Correspondence: mariagiovanna.chini@unimol.it (M.G.C.); casapullo@unisa.it (A.C.); Tel.: +39-0874404132 (M.G.C.); +39-089969243 (A.C.)

† These authors contributed equally to this work.

Table of contents

Figure S1. ^{1}H NMR ($CDCl_3$, 600 MHz) of *n*-hexane extract obtained from the modified Kupchan partitioning procedure of the hot water infusion (WI) of the inflorescences of *Cannabis sativa* L. *Kompolti* variety. Some selected ^{1}H NMR signals associated with CBD and CBDA have been evidenced. 3

Figure S2. ^{1}H NMR ($CDCl_3$, 600 MHz) of $CHCl_3$ extract obtained from the modified Kupchan partitioning procedure of the hot water infusion (WI) of the inflorescences of *Cannabis sativa* L. *Kompolti* variety. Some selected ^{1}H NMR signals associated with CBD and CBDA have been evidenced. 3

Figure S3. ^{1}H NMR (CD_3OD, 600 MHz) of *n*-BuOH extract (full (panel A) and enlarged (from 9.2 to 5.4 ppm) spectrum region (panel B)) obtained from the modified Kupchan partitioning procedure of the hot water infusion (WI) of the inflorescences of *Cannabis sativa* L. *Kompolti* variety. 4

Figure S4. ^{1}H NMR (CD_3OD, 600 MHz) of water extract (1D sequence without (panel A) and with (panel B) f1 presaturation and spectrum enlarged (from 9.2 to 5.0 ppm) region (panel C)) obtained from the modified Kupchan partitioning procedure of the hot water infusion (WI) of the inflorescences of *Cannabis sativa* L. *Kompolti* variety. 6

Figure S5. Chemical structures of Cannabidiol (CBD) and Cannabidiolic acid (CBDA). 7

Figure S6. ^{1}H NMR (CD_3OD, 400 MHz) of Cannabidiol (CBD). 8

^{1}H NMR assignments of CBD in CD_3OD. 8

Figure S7. ^{1}H NMR (CD_3OD, 400 MHz) of Cannabidiolic acid (CBDA). 9

^{1}H NMR assignments of CBDA in CD_3OD: 9

Figure S8. Alternative binding mode of CBD (colored by atom type: C blue, O red, polar H white) in complex with the three-dimensional structure of Keap1-Nrf2 peptide complex (red loop). H bond is reported in yellow 10

References 10

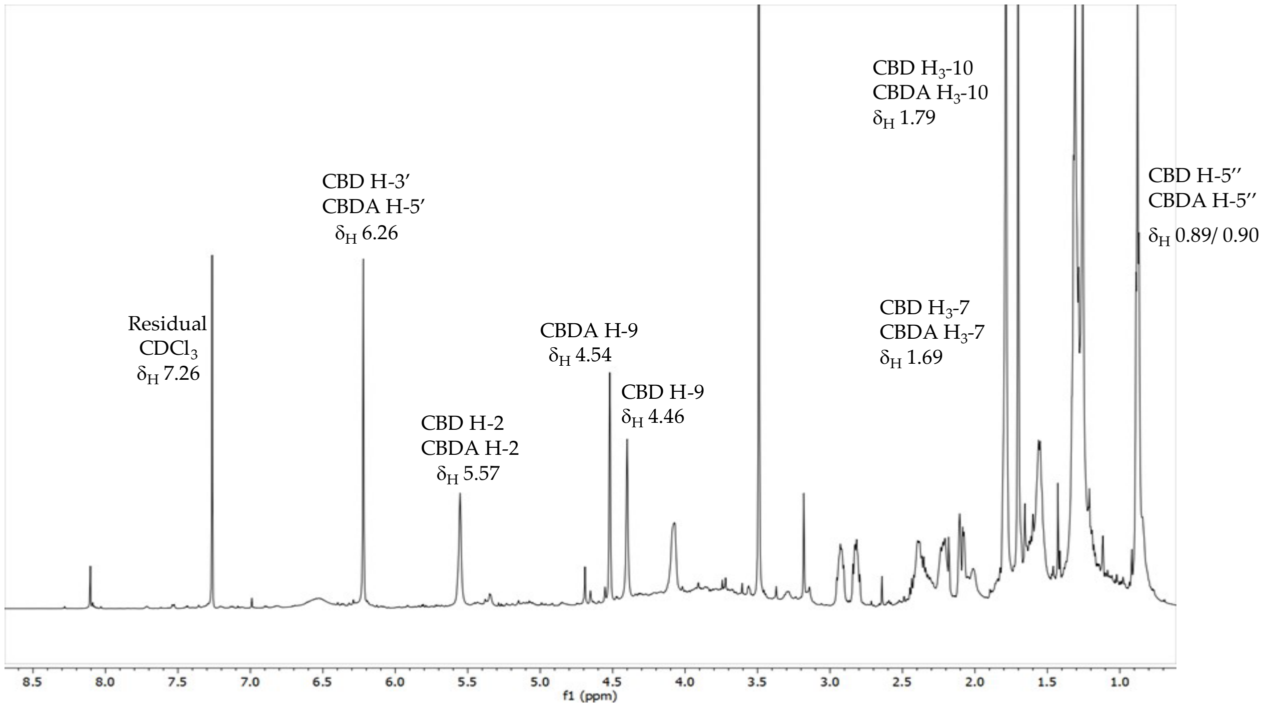

Figure S1. ^{1}H NMR ($CDCl_3$, 600 MHz) of *n*-hexane extract obtained from the modified Kupchan partitioning procedure of the hot water infusion (WI) of the inflorescences of *Cannabis sativa* L. *Kompolti* variety. Some selected ^{1}H NMR signals associated with CBD and CBDA have been evidenced [1].

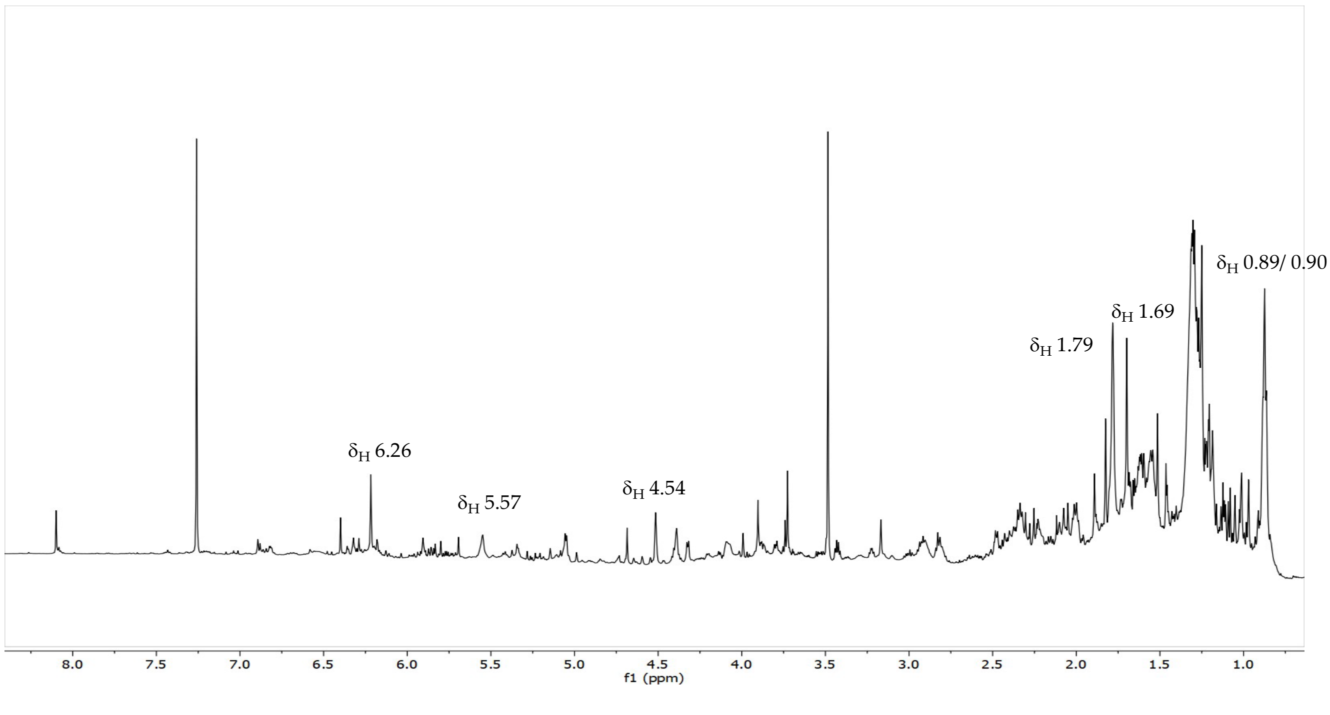

Figure S2. ^{1}H NMR ($CDCl_3$, 600 MHz) of $CHCl_3$ extract obtained from the modified Kupchan partitioning procedure of the hot water infusion (WI) of the inflorescences of *Cannabis sativa* L. *Kompolti* variety. Some selected ^{1}H NMR signals associated with CBD and CBDA have been evidenced.

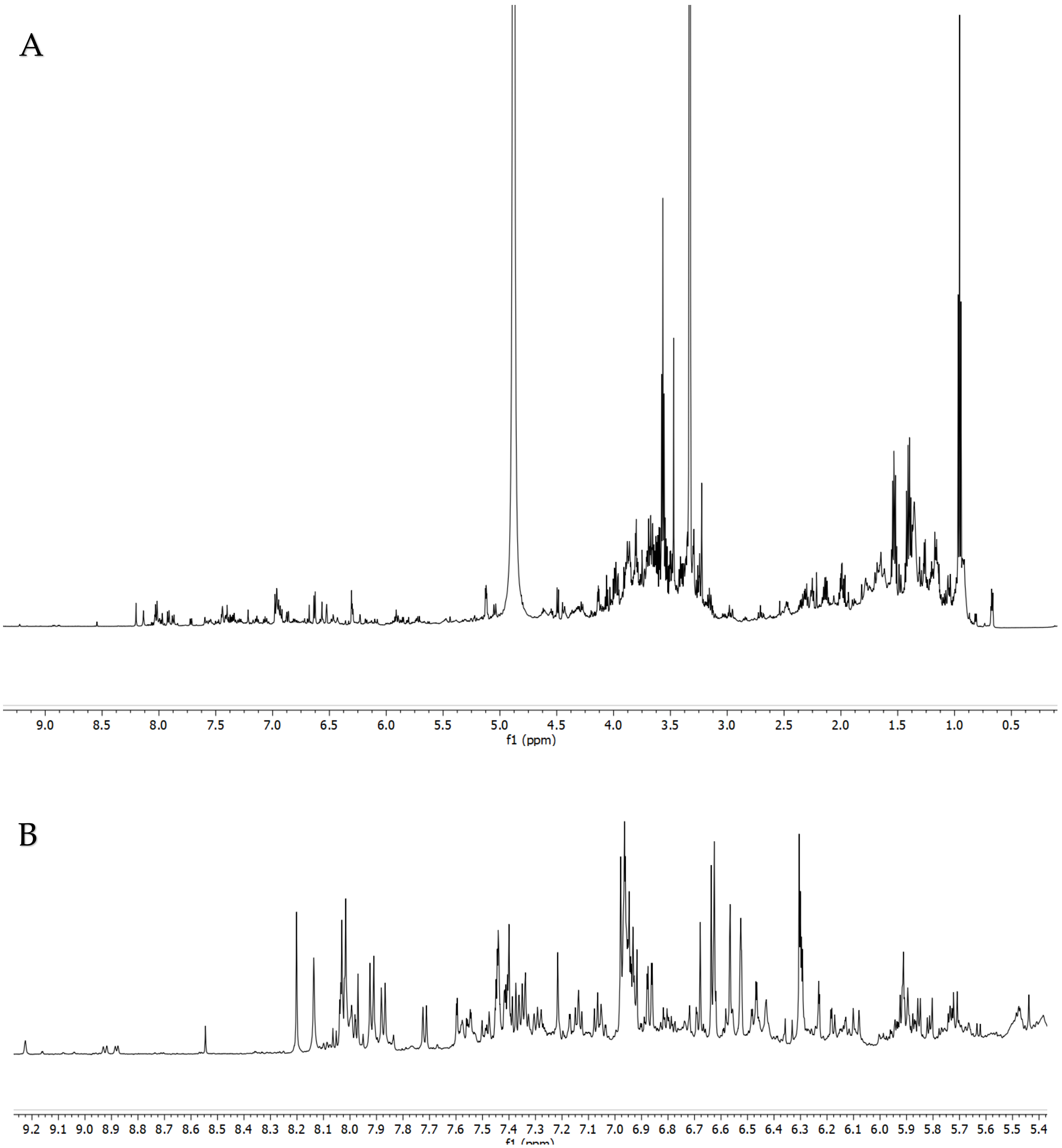

Figure S3. ^{1}H NMR (CD_3OD, 600 MHz) of *n*-BuOH extract (full (panel A) and enlarged (from 9.2 to 5.4 ppm) spectrum region (panel B)) obtained from the modified Kupchan partitioning procedure of the hot water infusion (WI) of the inflorescences of *Cannabis sativa* L. *Kompolti* variety.

A

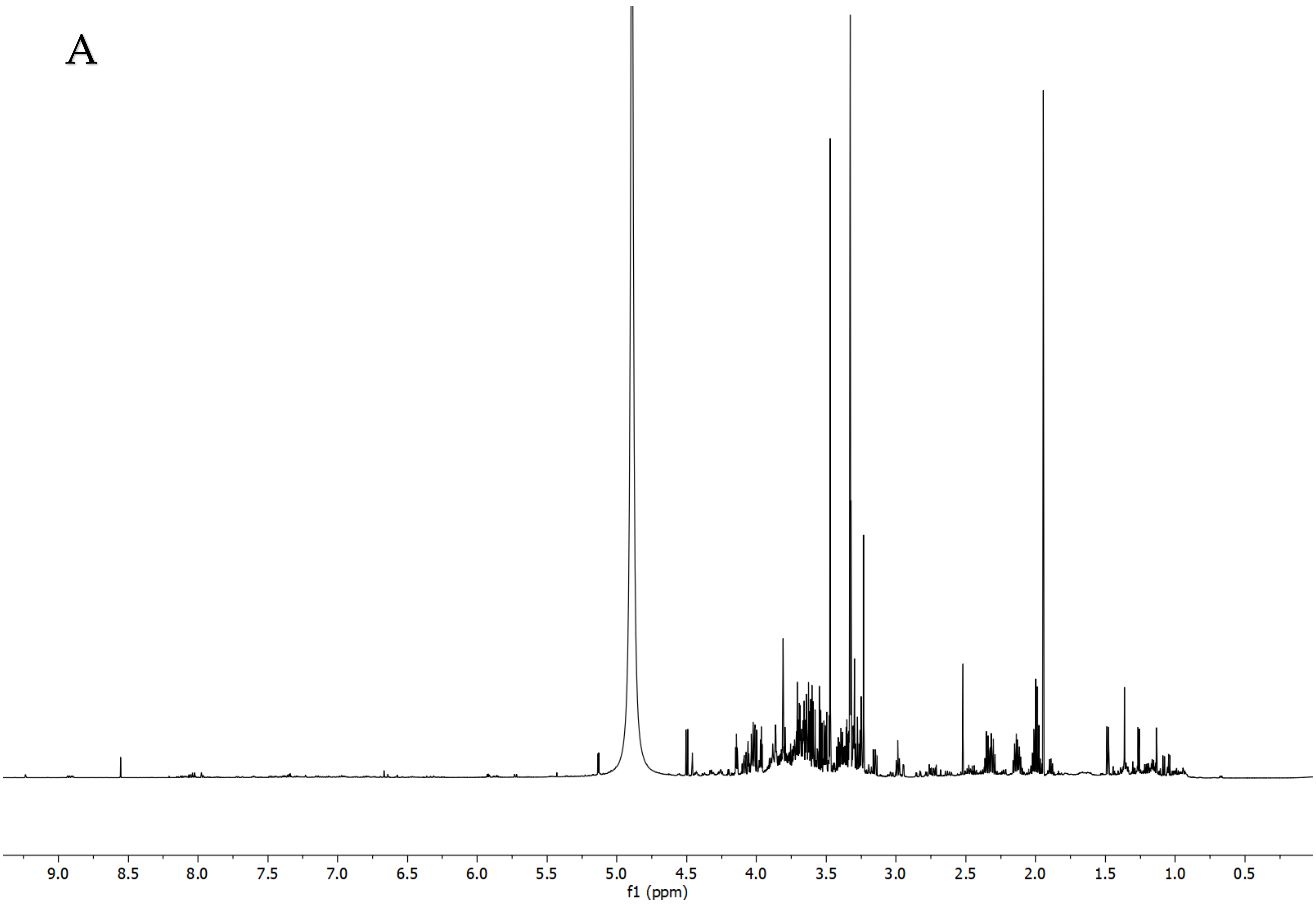
9.0 8.5 8.0 7.5 7.0 6.5 6.0 5.5 5.0 4.5 4.0 3.5 3.0 2.5 2.0 1.5 1.0 0.5
f1 (ppm)

B

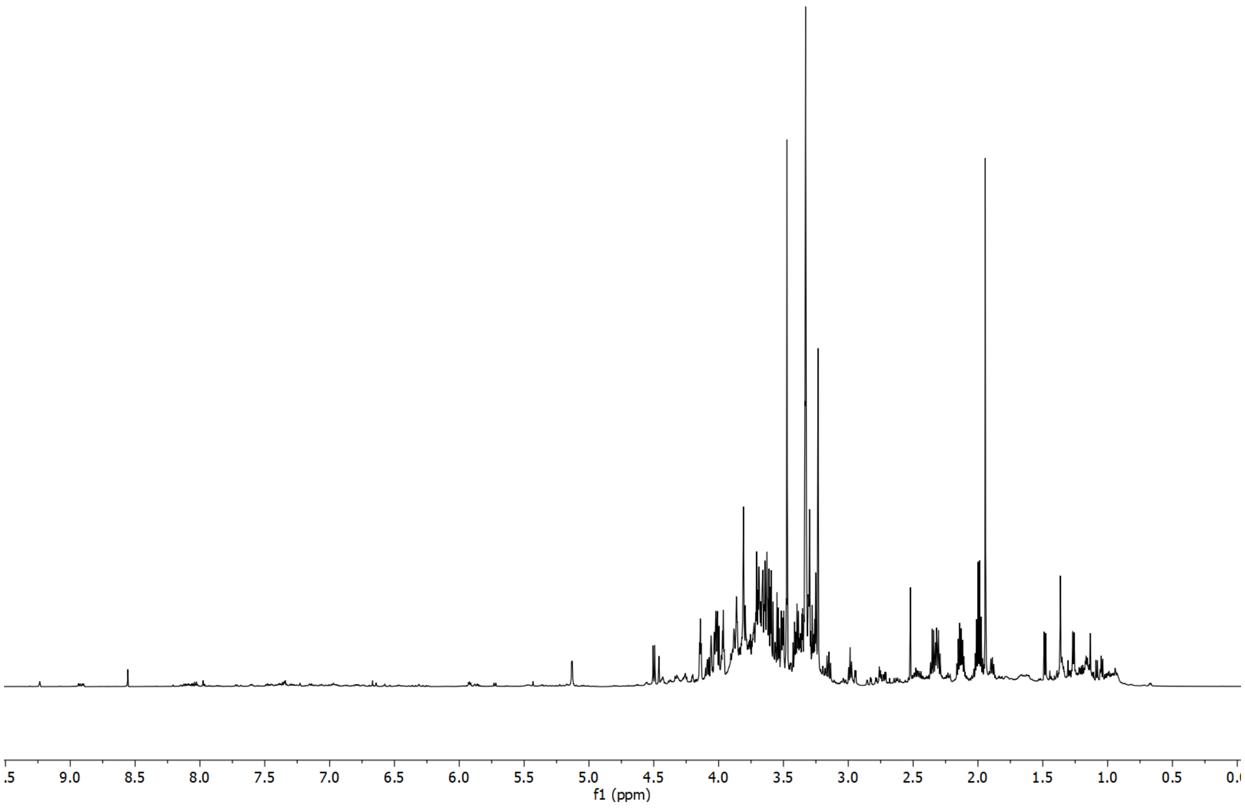
9.0 8.5 8.0 7.5 7.0 6.5 6.0 5.5 5.0 4.5 4.0 3.5 3.0 2.5 2.0 1.5 1.0 0.5
f1 (ppm)

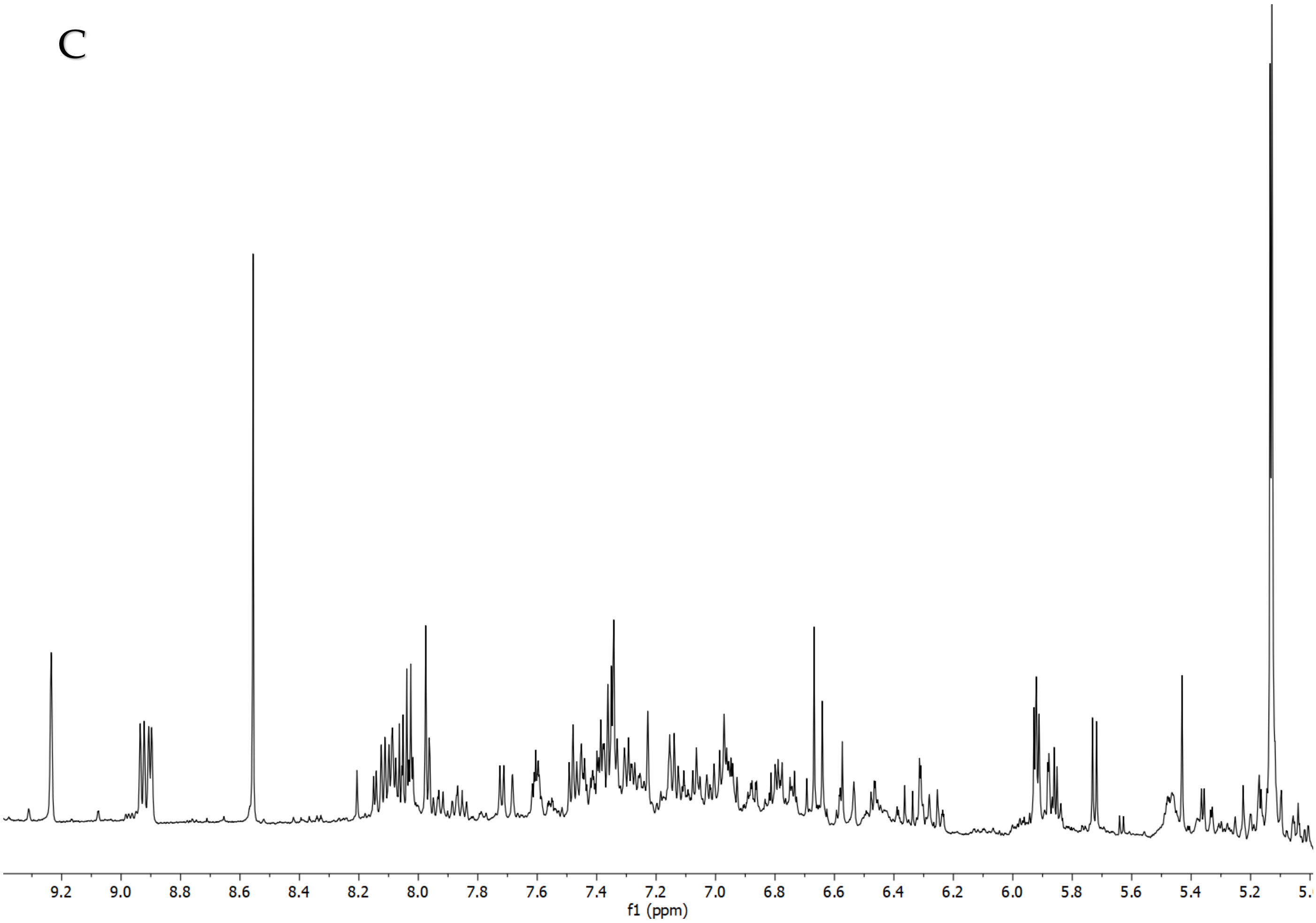

Figure S4. ^{1}H NMR (CD_3OD, 600 MHz) of water extract (1D sequence without (panel A) and with (panel B) f1 presaturation and spectrum enlarged (from 9.2 to 5.0 ppm) region (panel C)) obtained from the modified Kupchan partitioning procedure of the hot water infusion (WI) of the inflorescences of *Cannabis sativa* L. *Kompolti* variety.

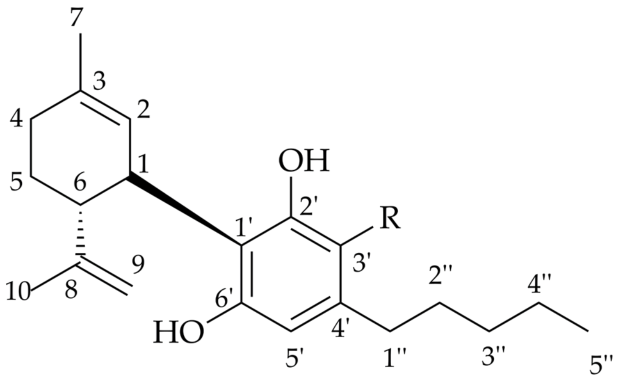

R = H, cannabidiol (CBD)
R = COOH, cannabidiolic acid (CBDA)

Figure S5. Chemical structures of Cannabidiol (CBD) and Cannabidiolic acid (CBDA).

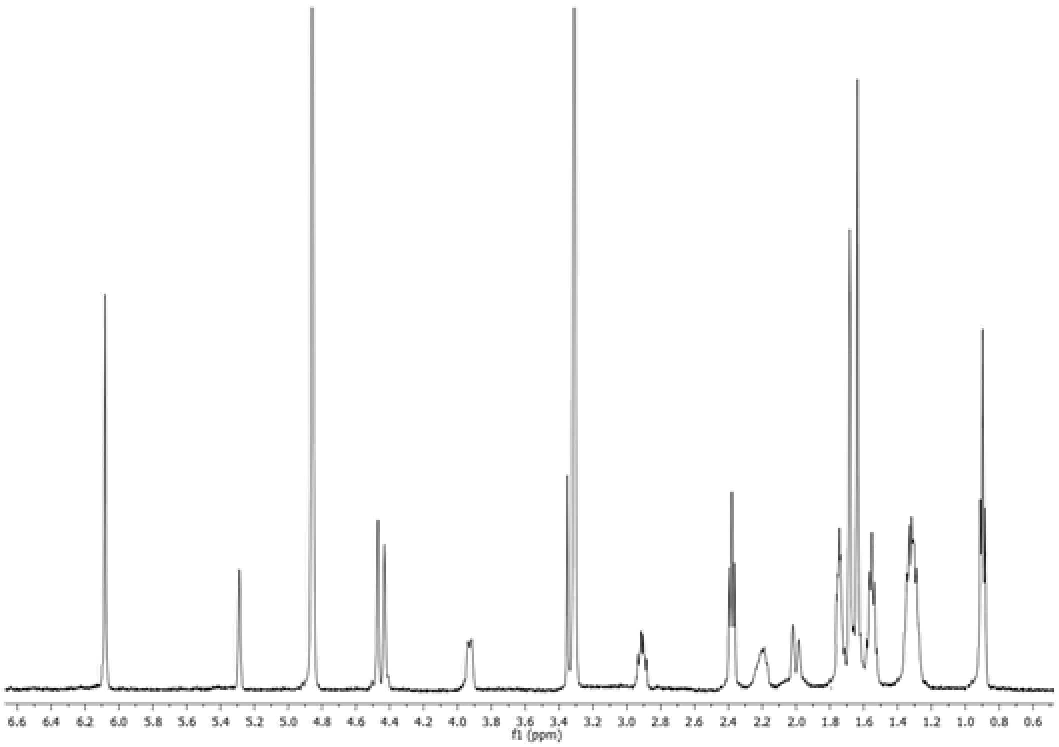

Figure S6. ^{1}H NMR (CD_3OD, 400 MHz) of Cannabidiol (CBD).

^{1}H NMR assignments of CBD in CD_3OD.

Cannabidiol CBD: ^{1}H NMR (400 MHz) data identical to those previously reported in literature [1]:

^{1}H NMR chemical shift data in CD_3OD: δ_H 6.07 (2H, brs, H-3′ and H-5′), 5.28 (1H, s, H-2), 4.46 and 4.42 (each 1H, m, H_2-9), 3.93 (1H, brd, 11.0 Hz, H-1), 2.89 (1H, m, H-6), 2.37 (2H, t, 7.5 Hz, H_2-1″), 2.18 and 1.99 (each 1H, m, H_2-4), 1.74 (2H, m, H_2-5), 1.67 (3H, s, H_3-7), 1.63 (3H, s, H_3-10), 1.53 (2H, m, H-2″), 1.29 (4H, m, H_2-3″ and H_2-4″), 0.89 (3H, t, 6.8 Hz, H_3-5″).

Chemical shifts are referenced to residual CHD_2OD (3.31 ppm) in CD_3OD; the number of protons, multiplicity, and coupling constants are shown in brackets.

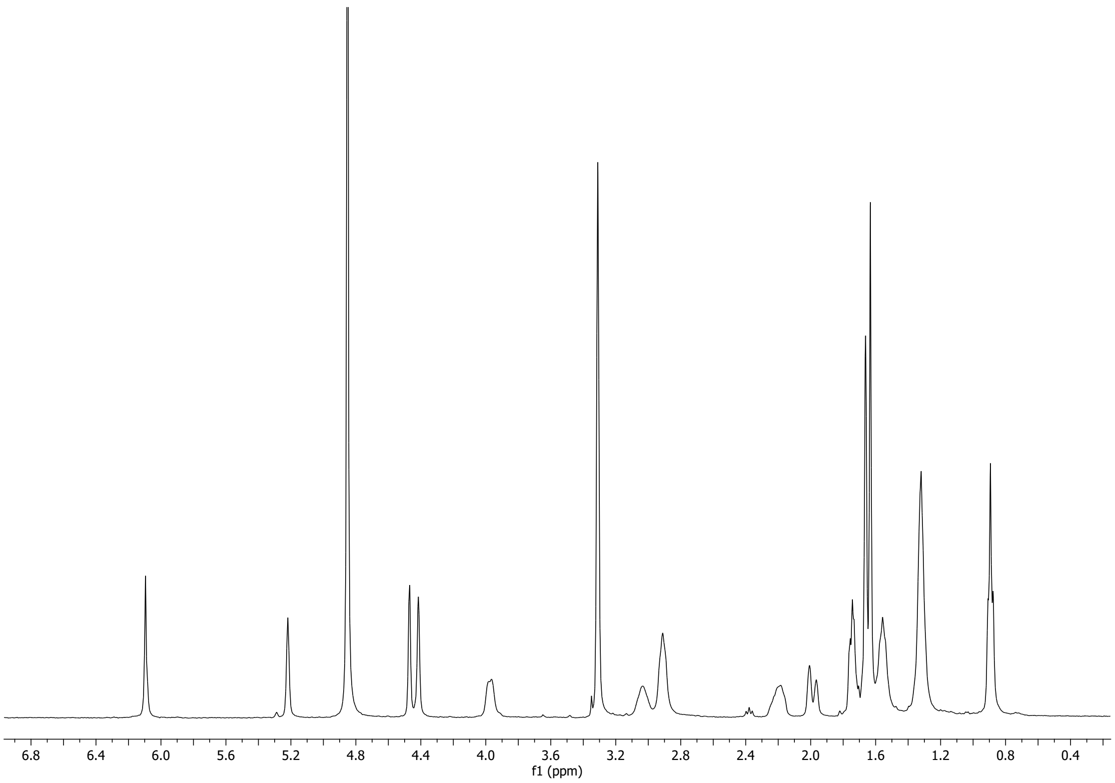

Figure S7. ^{1}H NMR (CD_3OD, 400 MHz) of Cannabidiolic acid (CBDA).

^{1}H NMR assignments of CBDA in CD_3OD:

Cannabidiolic acid CBDA: ^{1}H NMR (400 MHz) data identical to those previously reported in literature [1]: ^{1}H NMR chemical shift data in CD_3OD: δ_H 6.13 (1H, s, H-5′), 5.28 (1H, s, H-2), 4.45 and 4.42 (each 1H, m, H_2-9), 3.97 (1H, brd, 11.0 Hz, H-1), 3.03 (1H, m, H-6), 2.82 (2H, t, 7.5 Hz, H_2-1″), 2.19 and 1.98 (each 1H, m, H_2-4), 1.78 (2H, m, H_2-5), 1.65 (3H, s, H_3-7), 1.62 (3H, s, H_3-10), 1.58 (2H, m, H-2″), 1.31 (4H, m, H_2-3″ and H_2-4″), 0.89 (3H, t, 6.8 Hz, H_3-5″).

Chemical shifts are referenced to residual CHD_2OD (3.31 ppm) in CD_3OD; the number of protons, multiplicity and coupling constants are shown in brackets.

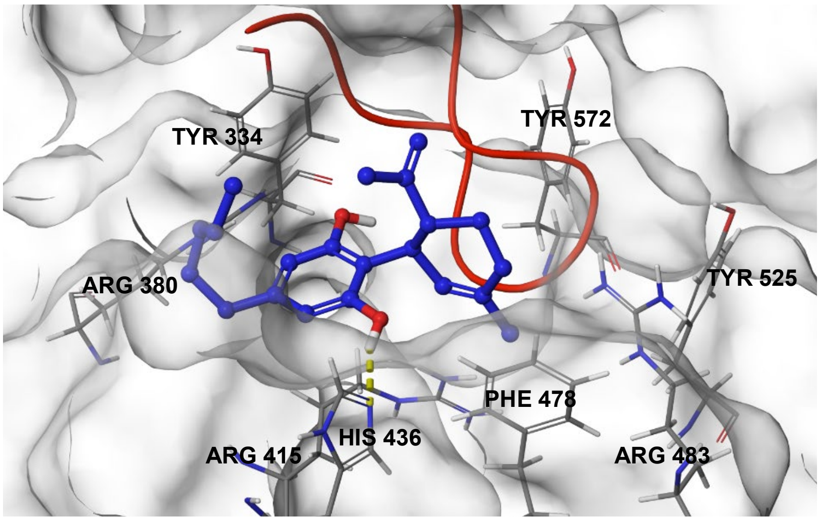

Figure S8. Alternative binding mode of CBD (colored by atom type: C blue, O red, polar H white) in complex with the three-dimensional structure of Keap1-Nrf2 peptide complex (red loop). H bond is reported in yellow.

References

1. Choi, Y.H.; Hazekamp, A.; Peltenburg - Looman, A.M.G.; Frédérich, M.; Erkelens, C.; Lefeber, A.W.M.; Verpoorte, R. NMR assignments of the major cannabinoids and cannabiflavonoids isolated from flowers of *Cannabis sativa*. *Phytochemical Analysis* **2004**, *15*, 345-354, doi:10.1002/pca.787.
